# The Longest Month: Analyzing COVID-19 Vaccination Opinions Dynamics From Tweets in the Month Following the First Vaccine Announcement

**DOI:** 10.1109/ACCESS.2021.3059821

**Published:** 2021-02-16

**Authors:** Liviu-Adrian Cotfas, Camelia Delcea, Ioan Roxin, Corina Ioanăş, Dana Simona Gherai, Federico Tajariol

**Affiliations:** 1 Department of Economic Informatics and CyberneticsBucharest University of Economic Studies 125536 010552 Bucharest Romania; 2 ELLIADD LaboratoryUniversity of Bourgogne Franche-Comté 25200 Montbéliard France; 3 Department of Accounting and AuditBucharest University of Economic Studies 125536 010552 Bucharest Romania; 4 Department of Finance-AccountingUniversity of Oradea 112936 410087 Oradea Romania

**Keywords:** Opinion mining, social media, COVID-19, SARS-CoV-2, stance classification, vaccine

## Abstract

The coronavirus outbreak has brought unprecedented measures, which forced the authorities to make decisions related to the instauration of lockdowns in the areas most hit by the pandemic. Social media has been an important support for people while passing through this difficult period. On November 9, 2020, when the first vaccine with more than 90% effective rate has been announced, the social media has reacted and people worldwide have started to express their feelings related to the vaccination, which was no longer a hypothesis but closer, each day, to become a reality. The present paper aims to analyze the dynamics of the opinions regarding COVID-19 vaccination by considering the one-month period following the first vaccine announcement, until the first vaccination took place in UK, in which the civil society has manifested a higher interest regarding the vaccination process. Classical machine learning and deep learning algorithms have been compared to select the best performing classifier. 2 349 659 tweets have been collected, analyzed, and put in connection with the events reported by the media. Based on the analysis, it can be observed that most of the tweets have a *neutral* stance, while the number of *in favor* tweets overpasses the number of *against* tweets. As for the news, it has been observed that the occurrence of tweets follows the trend of the events. Even more, the proposed approach can be used for a longer monitoring campaign that can help the governments to create appropriate means of communication and to evaluate them in order to provide clear and adequate information to the general public, which could increase the public trust in a vaccination campaign.

## I. Introduction

The coronavirus outbreak caused by the novel coronavirus SARS-CoV-2 has brought a series of changes in many aspects of people’s economic and social life. Since its occurrence, the coronavirus pandemic has continued to monopolize the different parts of the world, reaching 220 countries and territories by December 9, 2020 [1]. Governments have tried to address the outbreak by considering a series of measures, not all of them in accordance with the general public opinion. In all this time, the rapid growth of the number of cases globally has produced panic, fear and anxiety among people [2].

Due to the current situation generated by the lockdown in some parts of the world and social distancing in others, the use of social media globally has intensified [2], as it succeeds in connecting people from geographically different places and allows them to exchange ideas and information related to a series of aspects that have occurred in this period. Even more, people seem to rely on the information posted on social media. As a result, social media platforms have become mediator channels between each individual and the rest of the world and have gained more and more attention, being one of the fastest growing information systems for social applications [3], [4]. On this channel, individuals show their different views, opinions and emotions during the various events that occur due to the coronavirus pandemic [3].

Among some of the well-known social media platforms, Twitter has gained a particular attention as the users can easily broadcast information about their opinions on a given topic through a public message, called tweet [5]. Besides the information voluntarily offered by the user, a tweet may also retain information related to the location of the user and might contain links, emoticons and hashtags which can help the user in better expressing his/her sentiments, making it a source of valuable information [5], [6]. Even more, Twitter has been used by government officials and political figures for informing the general public either regarding their activity or in the case of major events occurrence [7].

Over time, the information extracted from Twitter has been used in various studies, featuring, but not being limited to: analyzing public opinion related to refugee crisis [8], natural disasters and social movements [9], evaluating companies’ services [10] and reputation [11], sports’ fans sentiments [12], [13], forecasting the prices of cryptocurrencies [14], predicting vehicle sales [15], political attitudes in multi-party contexts [16], healthcare [17], infectious disease [3], [18], celiac disease [19] and cancer patients sentiments [20], vaccination [5].

The vaccination topic has been, over time, one of the themes which have raised a series of questions in social media, most of them related to the safety of the entire process. As a result, a series of studies have analyzed the impact of different social media campaigns on vaccination hesitancy [21]–[23] or the general public sentiment in connection with the vaccination process [5], [24]. Additionally, compared to other vaccination situations studied in the scientific literature, the COVID-19 vaccination comes with new inquietudes related to the relatively short period of time needed for the vaccine development. As known, the process of developing a vaccine typically takes a decade [25]. Note, however, that the fastest vaccine development before has been four years [26] in the case of mumps vaccine and that, almost forty years after the discovery of HIV, no effective vaccine has yet been developed. However, the vaccine timelines for COVID-19 are reduced due to the emergency [25]. On December 18, 2020, the web site COVID-19 Vaccine Tracker,^1^ held by Milken Institute, shows 236 vaccines are in development, 38 are now in clinical testing and 7 have reached a regulatory decision. Nevertheless, on December 8, 2020 the first vaccine has been administrated in UK.^1^https://www.covid19vaccinetracker.org

In this context, the present paper analyzes the public opinion related to the vaccination process in the case of COVID-19, by considering the messages posted on Twitter. The period between November 9, 2020 – when Pfizer and BioNTech announced the development of a vaccine that is more than 90% effective, to December 8, 2020 – when the vaccination process has started in UK, has been considered. A number of 2 349 659 tweets have been collected and a cleaned dataset containing 752 951 tweets has been extracted. The performance for stance detection of several machine learning algorithms (both classical machine learning and deep learning algorithms) has been compared on an annotated dataset. The best performing algorithm has been selected and used for analyzing both the entire and the cleaned datasets.

The contribution of the paper is three-folded: we have collected and annotated a COVID-19 vaccination dataset, we have determined the best performing classifier for COVID-19 vaccination stance detection and we have put in relation the number of tweets and the stance (e.g. *in favor*, *against* or *neutral*) with the events reported by the media in the analyzed period.

The chosen approach can be easily integrated in a system which can allow interested organizations a proper monitoring of the public opinion regarding the vaccination process in the case of the new coronavirus.

The remainder of the paper is organized as follows. Section 2 provides a literature review structured in two main parts: natural language processing - focusing on sentiment analysis and stance detection from social media messages, and recent studies analyzing public opinion based on COVID-19 data extracted from Twitter. Section 3 describes the proposed methodology, while Section 4 focuses on the dataset collection and annotation process. Section 5 describes the steps required for stance detection and analyzes the performance of the classification algorithms. Section 6 presents the dynamics of opinions in the analyzed period. The limitations of the present study are mentioned in Section 7. The paper closes with a conclusion section and references. A series of supplementary materials accompany the paper, in the form of the collected and annotated datasets, along with the extracted unigrams, bigrams and trigrams for each day in the selected period.

## II. Literature Review

In the following, a short literature review regarding sentiment analysis and stance detection is conducted in order to underline the current approaches in the research literature. Afterwards, a series of studies that have analyzed the public opinion, in the context of the COVID-19 pandemic, using data extracted from Twitter are discussed.

### A. Sentiment Analysis and Stance Detection

Opinion mining is a growing area of the Natural Language Processing field commonly used to determine viewpoints towards targets of interest using computational methods [27]. It is also known as sentiment analysis and includes many sub-tasks, such as polarity detection – in which the goal is to determine whether a text has positive, negative or neutral connotation [28], emotion identification – in which the objective is to uncover specific emotions such as happiness, fear or sadness [29], subjectivity detection – in which the goal is to determine if the text is objective or subjective [30].

Stance detection [31], [32] is an opinion mining task used in debate analysis, for determining the opinions towards a specific target. It can be formalized as the task of identifying the tuple $< t,s>$, in which $t$ represents the target entity, while $s$ represents the opinion. The target entity ($t$) can be any discussion topic, including products, services, economic measures, or life choices, such as vaccination. The opinion ($s$) towards the target is identified as *in favor*, *against* or *neutral* [27].

While similar in some respects to polarity detection, stance detection is a different natural language processing task, given the fact that positive tweets can be against the target entity, while on the contrary, negative tweets can sometimes express a favorable view of the target entity. Moreover, when compared to polarity detection, stance detection always determines the agreement or disagreement in relation to a specific target, even in cases in which the target is not explicitly mentioned in the analyzed text [5].

The types of approaches that can be used for polarity analysis and stance detection include: lexicon-based methods [33], machine learning methods [34] and hybrid methods – in which lexicons and machine learning are combined [35], [36].

Lexicon based methods rely on sentiment lexicons, such as Bing Liu’s opinion lexicon [37], MaxDiff [38], Sentiment140 [39], VaderSentiment [40], SentiWordNet [41] or SenticNet [42], which contain words and sequences of words, together with the polarity score, indicating the strength of the positive, neutral or negative perception. For performing polarity detection, the sentiment lexicons are used together with semantic methods, which typically consider negations and booster words [40]. A simple rule-based model incorporating a sentiment lexicon, as well as grammatical and syntactical conventions, called Vader, is proposed by Hutto and Gilbert [40]. The authors show that the proposed model outperforms individual human raters. When compared to classical machine learning algorithms (such as Support Vector Machines, Naïve Bayes and Maximum Entropy), the authors show that Vader offers a better performance on the datasets collected from Twitter, Amazon reviews and NYT editorials. Given the fact that the creation of lexicons is time consuming, Cotfas *et al.* [33] have shown that multiple existing lexicons can be combined to create more comprehensive lexicons through the advantages brought by the grey systems theory. Compared to machine learning, lexicon-based approaches have the advantage of not requiring the collection and annotation of training data, making them preferable when the volume or the quality of the training data is not sufficient [43], [44].

Machine Learning approaches use supervised classification algorithms to extract knowledge regarding the sentiment polarity or the stance of a text. As a preliminary step, before applying machine learning, the text needs to be first converted into numerical vectors, using schemes such as Bag-of-Words and word embeddings. The Bag-of-Words approach is a flexible text representation scheme that describes the number of occurrences of words in the encoded document. As a disadvantage, this scheme does not consider the sequence in which the words appear in the document, thus ignoring the context in which they are used [45]. Word embeddings are a text representation approach in which each word is mapped to a vector having the values computed in such a way that allows words which frequently appear in similar contexts to have a similar representation [46]. The main benefit of this representation is that additional clues become available for the classification algorithms. Another advantage resides in the fact that the number of required dimensions is greatly reduced when compared to a sparse vector representation, such as one-hot encoding, in which each term is as a binary vector that contains only zeros, besides a single one-value, corresponding to the term’s index in the vocabulary [45]. Among the most popular word embedding techniques, one can mention: embedding layer, Word2Vec [47], GloVe [48] and FastText [49].

Machine learning approaches include classical machine learning and deep learning algorithms. Frequently used classical machine learning algorithms for stance detection are Support Vector Machines (SVM) [5], [31], [50] and Naïve Bayes (NB) [5]. In the context of the “SemEval-2016 Task 6: Detecting Stance in Tweets” [51], the SVM classifier with unigram features, used as a baseline for the algorithms developed by the competing teams, has achieved and F-Score of 63.31. By incorporating also word n-grams (unigrams, bigrams and trigrams) and character n-grams (with lengths {2, 3, 4, 5}) the F-Score has increased to 68.98, higher than all the scores recorded by the algorithms proposed during the competition [51]. D’Andrea *et al.* [5] have compared several classical machine learning (including SVM and NB) and deep learning algorithms for detecting the stance towards vaccination in Italian tweets, achieving the best results when using SVM. The approach proposed by D’Andrea *et al.* [5] has constituted the basis for the current study.

Deep Learning algorithms have become particularly popular in recent years for both stance detection [31] and sentiment analysis [52]. The Deep Learning based techniques have predominantly used Convolutional Neural Networks (CNN) [5], [53] and Recurrent Neural Networks (RNN) [54], [55], with its variant Long Short-Term Memory (LSTM) [5], [56]–[58]. Zarrella and Marsh [58] have proposed a LSTM approach that has achieved an F-Score of 67.82, one of the highest scores among the competing teams at “SemEval-2016 Task 6: Detecting Stance in Tweets”. However, the algorithm has performed worse than the baseline SVM n-grams algorithm.

As an alternative to RNN and CNN, Vaswani *et al.* [59] have proposed transformers, an attention-based architecture, replacing the recurrent layers with multi-headed self-attention, achieving state of the art results for machine translation [59], document generation [60] and syntactic parsing [61]. Transformer-based language models, pre-trained on large and diverse corpuses of unlabeled data, such as Generative Pre-trained Transformer (Open-AI GPT) [62] and Bidirectional Encoder Representations from Transformers (BERT) [63] can be afterwards easily fine-tuned for a wide range of Natural Language Processing (NLP) tasks [62], [63]. While Open-AI GPT uses a unidirectional left-to-right architecture, BERT relies on a bidirectional approach, providing better results on many NLP tasks, including sentiment analysis [63].

Hybrid methods feature a combination of lexicons and machine learning algorithms. Aloufi and Saddik [35] have performed polarity detection from football-specific tweets using several machine learning algorithms and a sentiment lexicon automatically generated starting from a manually labeled dataset. Even though some improvements have been noticed by the authors in comparison to using general lexicons, the best results have been achieved by SVM with unigrams.

Comparisons between various stance analysis approaches used in social media analysis are included in Wang *et al.* [31] and Mohammad *et al.* [51].

### B. Twitter Sentiment Analysis on COVID-19 Data

In the case of epidemics, Merchant and Lurie [64] have observed that besides the role assumed by social media of becoming the fastest channel of communication between people found in situations of social distancing due to lockdown, the social media can also act as a tool which can be used for anticipating the circumstances related to the spread of epidemics around the world. The authors have observed a high correlation between the information posted on Twitter regarding the evolution of an epidemic and the official data released by the Center for Disease Control and Prevention. As a result, the authors have concluded that Twitter can provide real-time estimations and predictions in the case of epidemic-related activities. Based on this research, Kaur *et al.* [65] have used the data extracted from Twitter to monitor the dynamics of emotions during the first months after the COVID-19 has become known to the public. A total number of 16 138 tweets have been extracted and analyzed using IBM Watson Tome Analyzer. As expected, the number of negative tweets exceeded the number of neutral and positive tweets in all the three months considered in the paper. Comparing the sentiments extracted for June with the ones extracted for February, it has been observed that the proportion of negative sentiments has decreased (from 43.92% to 38.05%), while the positive sentiments proportion has increased (from 21.38% to 27.01%). The proportion of the neutral sentiments has been almost the same (34.07% in February vs. 34.94% in June).

The prevalence of negative sentiments over the positive ones in the case of the COVID-19 pandemic has been also underlined by Singh *et al.* [66], while Boon-Itt and Skunkan [67] have recorded a high discrepancy between the negative sentiments (covering 77.88% of tweets) and the positive sentiments (covering the rest of 22.12%).

Xue *et al.* [68] have analyzed the public sentiment related to 11 selected topics determined using Latent Dirichlet Allocation on COVID-19 tweets. The authors have concluded that fear is the most dominant emotion in all the considered topics and that the findings are in line with other studies on COVID-19 which state that human psychological conditions are significantly impacted by the coronavirus outbreak [68].

On the other hand, Bhat *et al.* [69] found that the most prominent sentiment was positive in the analysis conducted in their paper. The authors state that the occurrence of the positive sentiments in 51.97% of tweets can be a sign that the users who have posted the messages are hopeful and enjoy the socialization experience shared with the family in this period of lockdown and limited social interaction.

At regional level, Kruspe *et al.* [70] have analyzed geotagged tweets in Europe regarding COVID-19 through the use of a neural network, featuring a multilingual version of BERT, which has been trained on an external dataset, not connected to the COVID-19 outbreak. Based on their results, the authors state that they have observed a general downward trend of the negative sentiments as the time passes.

At national level, several studies have been conducted for different countries around the world. For example, in a study conducted on tweets extracted for Nepal, Pokharel [71] observed that the public opinion faced positive sentiments (58% of the tweets), while the negative sentiments have only been expressed in 15% of the tweets. The study used a Naïve Bayes model applied on a limited number of tweets (615 tweets). Barkur *et al.* [72] determined that in the case of the tweets from India, the positive sentiment was dominant when analyzing the national lockdown situation announced by the government. Similar conclusions have been reached by Khan *et al.* [73] in a research that has used Naïve Bayes classifier. The difference between the reactions towards the pandemic in different cultures has been studied by Imran *et al.* [74] through sentiment and emotion analysis, implemented with deep learning classifiers. Besides the correlation between tweets’ polarity from different countries, the authors also state that NLP can be used to link the emotions expressed on social platforms to the actual events during the coronavirus pandemic. Samuel *et al.* [75] have shown insights related to the evolution of the fear-sentiment over time in the United States.

At regional level, Zhou *et al.* [76] analyzed the sentiments in local government areas located in Australia and found that the general sentiment during the COVID-19 pandemic was a positive one, but there have been observed decreases in the positive polarity as the pandemic advanced, with significant changes from positive to negative sentiments depending on the government policies or social events. Wang *et al.* [77] made a comparative analysis between the tweets posted in California and New York and concluded that California had more negative sentiments than New York and that the fluctuation in sentiment scores can be correlated with the severity of COVID-19 pandemic and policy changes. Pastor [78] analyzed the sentiment of the Filipinos located in Luzon area and concluded that most Filipinos had negative sentiments, most of them due to the extreme community quarantine.

Some other analyses on Twitter in the context of COVID-19 have focused, but have not been limited to: topical sentiment analysis regarding the use of masks [79], monitoring depression trends [80], sentiment dynamics related to cruise tourism [81], identifying discussion topics and emotions [82], thematic analysis [83], detecting misleading information [84].

As shown above, the prominent sentiments related to COVID-19 have been found to be either positive or negative. The expressed sentiments have been shown to depend on the geographic area, government decisions and number of recorded cases. A more in-depth analysis related to the studies on sentiment analysis featuring COVID-19 and other infectious diseases can be found in Alamoodi *et al.* [3].

In this context, the present paper aims to analyze the stance of the Twitter users in connection to the new upcoming vaccines for COVID-19 in the first month after Pfizer and BioNTech announced their results on the new vaccine. The methodology and data collection process are presented in the following sections.

## III. Methodology

**FIGURE 1.**
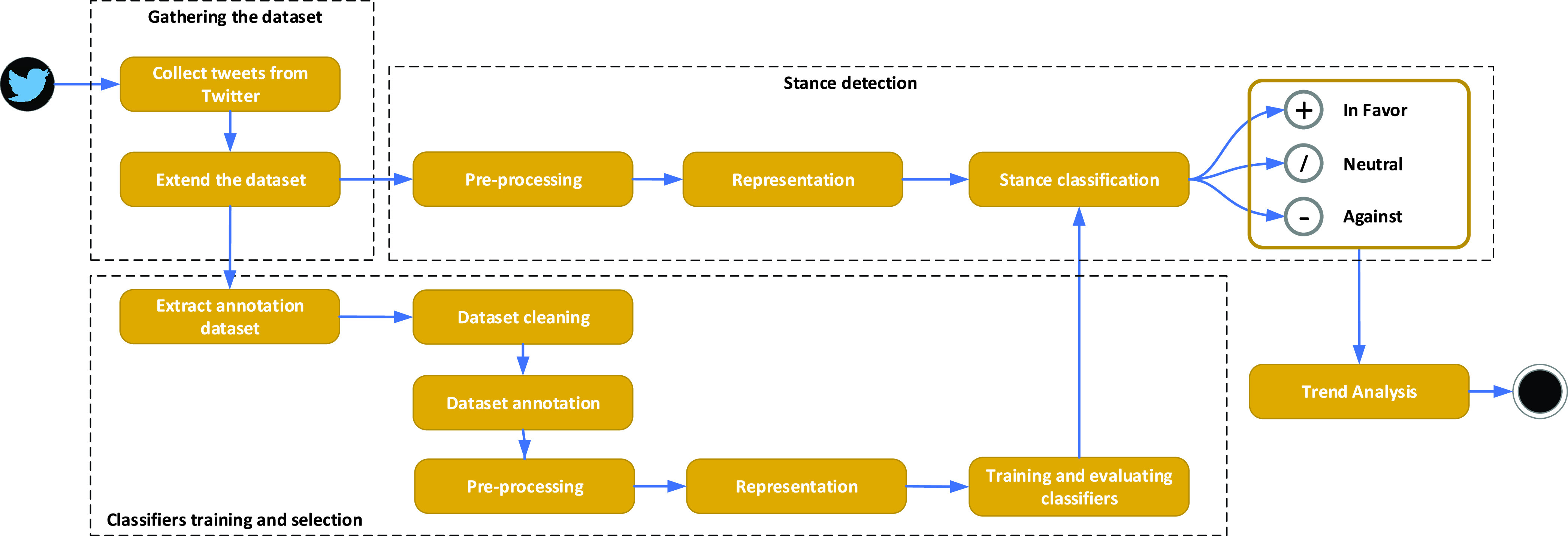
Steps of the proposed stance-detection approach.

The steps taken in order to analyze the public’s opinion regarding COVID-19 vaccination from social media messages are shown in Figure 1.

The initial step is to collect a COVID-19 vaccination stance dataset containing English language tweets. A randomly sampled subset from this dataset has been afterwards manually annotated as *neutral*, *in favor*, or *against* vaccination, in order to be used in the training phase of the stance classification algorithms.

Given their unstructured nature and informal writing style, in the following step, the tweets from the collected dataset have been pre-processed, with the purpose of improving the performance of the stance classification algorithms.

For text representation and classification, four approaches have been investigated: 1) Bag-of-Words representation followed by classical machine learning, 2) Word embeddings followed by classical machine learning, 3) Word embeddings followed by deep learning and 4) Bidirectional Encoder Representations from Transformers.

In order to determine the best performing classification algorithm, the text has been represented using both Bag-of-Words and word embeddings schemes. In the present paper, the performance of multiple classical machine learning and deep learning algorithms has been evaluated based on the following widely used metrics: Accuracy, Precision, Recall and F-score. Accuracy, which indicates the ratio of correctly predicted observations to the total observations is defined as shown in (1), in which TP, TN, FP and FN refer to true positive, true negative, false positive and false negative. Thus, TP represents the number of real positive tweets classified as positive, FP is the number of real negative tweets classified incorrectly classified as positives, TN represents the number of negative tweets correctly classified as negative and FN is the number of real positive tweets incorrectly classified as negative.

\begin{equation*} \mathrm {Accuracy}=\frac {\mathrm {TP}+\mathrm {TN}}{\mathrm {TP}+\mathrm {TN}+\mathrm {FP}+\mathrm {FN}}\tag{1}\end{equation*}

Precision, which represents the ratio of correctly predicted positive observations to the total predicted positive observations, is computed as shown in (2).

\begin{equation*} \mathrm {Precision}=\frac {\mathrm {TP}}{\mathrm {TP}+\mathrm {FP}}\tag{2}\end{equation*}

Recall, representing the ratio of correctly predicted positive observations to all the observations in the actual class, is computed as shown in (3).

\begin{equation*} \mathrm {Recall}=\frac {\mathrm {TP}}{\mathrm {TP}+\mathrm {FN}}\tag{3}\end{equation*}

Starting from Precision and Recall, the F-Score can be computed as a weighted average, as shown in (4).

\begin{equation*} \mathrm {F-score}=2\cdot \frac {\mathrm {Precision}\cdot \mathrm { Recall}}{\mathrm {Precision}+\mathrm {Recall}}\tag{4}\end{equation*}

Finally, the best performing algorithm has been used to analyze the evolution of the public stance towards vaccination in the considered period. The evolution has been correlated with the major events and news that have followed the announcement of the Pfizer and BioNTech vaccine results.

## IV. COVID-19 Vaccine Stance Dataset

A machine learning approach has been chosen for detecting the stance of the tweets, which requires a labeled dataset for training the classification models. Since we have not identified an already labeled dataset for stance towards COVID-19 vaccination in the scientific literature, a domain-specific dataset, having Twitter as a data source, has been collected and manually annotated. It should be also mentioned, that according to [31], there is a general lack of annotated corpuses for stance detection.

### A. Dataset Collection

Several public datasets including large-scale collections of tweets related to the coronavirus pandemic have been proposed in the scientific literature, including the ones presented in [85]–[88]. Some of the datasets, such as [86], [88], are multi-lingual, while others, such as [85], [87] are language specific, including only tweets written in English.

In order to collect a dataset centered around COVID-19 vaccination, a hybrid approach has been chosen, in which the tweets that we have fetched through the Twitter API for the keywords in Table 1, have been supplemented with the ones in the dataset described in [86], selected using the same keywords.

**TABLE 1:** Set of Keywords Used to Fetch Tweets

Topic	Keywords
covid-19	covid19, covid-19, coronavirus, coronaoutbreak, coronaviruspandemic, wuhanvirus, 2019nCoV
vaccination	vaccine, vaccination, vaccinate, vaccinating, vaccinated

Gathering the tweets from the Twitter API has been performed through the Twitter Filtered Stream API, with the help of the TweetInvi^2^ library.^2^https://github.com/linvi/tweetinvi

While the approach proposed in this paper can be extended to other languages, in the present study only tweets written in English have been considered. Thus, between November 9 and December 8 a number of 2 349 659 tweets concerning the topic of COVID-19 vaccination have been identified.

### B. Dataset Annotation

To ensure the quality of the annotated dataset, that will be used for training the machine learning algorithms, duplicated tweets have been discarded, as well as retweets. The retweets have been easily identified due to the presence of the “RT” symbol. This choice is in accordance with the approach from other studies, including, but not limited to [5], [35]. The remaining number of tweets in the cleaned dataset is 752 951, representing 32.04% of the initial dataset. Table 2 includes for each day in the considered period both the total number of tweets, as well as the remaining number of tweets after the duplicates and retweets have been eliminated.

**TABLE 2:** Number of Vaccine Related Tweets Published in the Considered Period

Date	Nov. 9	Nov. 10	Nov. 11	Nov. 12	Nov. 13	Nov. 14	Nov. 15	Nov. 16	Nov. 17	Nov. 18
Tweets	57 265	109 839	85 901	75 341	72 512	41 387	41 335	159 611	79 275	110 131
Cleaned	56 768	29 693	22 583	19 690	19 854	11 103	11 873	38 072	23 814	28 148
Date	Nov. 19	Nov. 20	Nov. 21	Nov. 22	Nov. 23	Nov. 24	Nov. 25	Nov. 26	Nov. 27	Nov. 28
Tweets	58 878	59 674	37 816	44 717	99 690	62 186	44 621	35 807	55 701	52 977
Cleaned	19 530	21 036	12 733	13 940	31 514	22 300	15 246	12 236	15 425	13 469
Date	Nov. 29	Nov. 30	Dec. 1	Dec. 2	Dec. 3	Dec. 4	Dec. 5	Dec. 6	Dec. 7	Dec. 8
Tweets	35 497	82 639	51 407	154 004	135 162	97 410	69 856	64 438	122 134	216 822
Cleaned	11 704	21 697	19 719	49 589	45 288	32 345	21 428	20 104	31 851	60 199

From the cleaned dataset we have randomly selected and manually annotated 7530 tweets, representing approximately 1.00% of all the tweets in the dataset. The number is higher than the one used in other stance detection approaches, such as D’Andrea *et al.* [5] and Mohammad *et al.* [89]. D’Andrea *et al.* [5] have trained the algorithms on a manually labeled dataset containing 693 tweets. The dataset proposed by Mohammad *et al.* [89] is organized on several topics, with the largest topic numbering 984 tweets.

In the present approach, the stance of the tweets towards vaccination has been evaluated by three independent human raters into three classes: *in favor*, *against* and *neutral*. Disagreements between the annotated tweets have only been recorded between the *in favor* and *neutral* or between the *neutral* and *against* stances. No disagreement has been recorded between *in favor* and *against* annotations. In the case of disagreement, the class chosen by most annotators has been associated with the tweet.

The distribution of the tweets in the annotated dataset in the three considered categories is illustrated in Table 3.

**TABLE 3:** Statistics for the Manually Annotated Dataset

Class	Number	Percent
against	1083	14.38%
neutral	5188	68.90%
in favor	1259	16.72%
Total	7530	100.00%

Tweets that have been assigned to the class *in favor* express a positive opinion regarding the vaccination. Tweets belonging to the *against* vaccination class express a negative opinion towards COVID-19 vaccination. The *neutral* class mainly includes news related to the development of vaccines, tweets that do not express a clear opinion, such as questions regarding the vaccine, informative tweets concerning vaccination, as well as off-topic tweets, many of them related to the 2020 presidential election in the United States, which was held nominally, just a few days before the analyzed period, on November 3, 2020. Several examples of manually labeled tweets belonging to the three categories are included in Table 4.

**TABLE 4:** Examples of Tweets Against, Neutral and in Favor

Stance	Tweet
against	WHY would i take a vaccine for a bug that the CDC says i have a 99% chance against, with just my God given immune system? I have a bigger chance of dying from a car accident or a bee sting. https://t.co/ylGR1HhzLe
	Do you want to be a guinea pig for a virus that 97% of people survive? Why is there even a vaccine for an illness with such a high survival rate? https://t.co/TsaMidiABr
	No way!!! Over my dead body, I won’t have a vaccine in my body, why would I.. I’m healthy!!!! #NoVaccine #FreedomFirst #mybodymychoice https://t.co/GQ9VFHPbjZ
	People cheering for the nano tech infested vaccines. Humanity has reached a level of stupidity that is truly mind blowing https://t.co/uUj29ejXtY
neutral	Italy will receive an initial 3.4 mln shots of the Pfizer coronavirus vaccine,probably in January,a government source told Reuters on Tuesday. The country will be allocated some 13.6% of the first 200 million doses made available to Europe, the source said.https://t.co/LftlQ1WYmn https://t.co/ueeH407K4I
	So how does the Pfizer Covid-19 vaccine injection work? Do they inject it at −90 degrees F. ?
	#Covid19 vaccine is here https://t.co/xK3UMSENN1
	Airlines scramble to prepare for ultra-cold COVID-19 vaccine distribution https://t.co/isvziZG0hi https://t.co/OATFux2Npq
in favor	Omg 90% effective coronavirus vaccine ???? Light at the end of the tunnel ! so you’re telling me there a CHANCE that I can actually walk graduation
	All I want for Christmas is covid19 vaccine. https://t.co/WKThNcYWYr
	Kudos to all the scientists and medical experts working tirelessly to produce a safe and productive vaccine to defeat COVID-19
	I want someone to find a damn vaccine or cure for this STUPID VIRUS man. Tired of seeing it affect families and kill ppl. Tired of walking past the ICU of my job seeing folks fighting for their lives. Sick of these lingering effects from me having it. SICK OF COVID-19

The n-grams and balanced annotated dataset are available at the following link: https://github.com/liviucotfas/covid-19-vaccination-stance-detection

## V. COVID-19 Vaccination Stance Detection

The main components of the stance detection process are the pre-processing, the feature extraction and the machine learning classification. In the pre-processing step the text is cleaned, while in the feature extraction the raw textual data is converted to feature vectors. The classical machine learning and deep learning classifiers, that have been compared in this paper, are described within this section.

### A. Pre-Processing

Given the fact that social media messages are frequently written using a casual language, a pre-processing step has been used in order to prepare the tweets in the annotated dataset for training the machine learning classifiers. This step is considered crucial by D’Andrea *et al.* [5] for the success of the entire system, while Bao *et al.* [90] provide a comprehensive discussion regarding the importance of pre-processing in social media analysis. The impact of the different pre-processing steps, such as the removal of links, on the performance of classical machine learning classifiers has been discussed by Jianqiang and Xiaolin [91].

During this pre-processing step, all the user mentions, easily identified through the presence of the @ symbol at the beginning of the message have been normalized, since they do not provide any useful information for the classification process. All the links and email addresses have been normalized as well. The emoticons have been replaced with the corresponding words. Minor spelling mistakes have been automatically corrected to improve performance. Contractions and hashtags have been unpacked, while elongated words have been corrected and annotated. Finally, all the letters have been converted to a lowercase representation. The pre-processing has been implemented with the help of the ekphrasis library [92]. Additional processing has been performed through Natural Language Toolkit (NLTK) library [93] and the “re” python module.

### B. Features

In order to use machine learning algorithms for text classification, the text content has to be first converted into numerical feature vectors. The Bag-of-Words (BoW) scheme converts the text to a numerical representation, having as a starting point the frequency of the words. Given a vocabulary $V=\{w_{1},\ldots,w_{N}$, containing $N$ tokens, denoted using $w_{i}$, a tweet, or any other textual document $d$, belonging to a corpus $D$, can be represented using a feature vector $X=\{x_{1},..,x_{N}$, in which $x_{i}$ can either represent a binary variable that indicates whether the word $w_{i}$ appears in the text or a numeric variable indicating the number of times the word $w_{i}$ appears in the text.

Given the fact that very frequent words can sometimes carry little “informational content”, the performance of classification algorithms that rely on word frequencies can be improved using a more complex feature representation, called Term Frequency - Inverse Document Frequency (TF-IDF), that reduces the weight associated to words that frequently appear in all the documents in the corpus. TF-IDF is computed as shown in (5):

\begin{equation*} TF-{IDF}_{(w_{i})}={TF}_{(w_{i})}\times log\frac {\left |{ D }\right |}{DF_{(w_{i})}}\tag{5}\end{equation*}

where ${\mathrm {TF}}_{(w_{i})}$ represents the number of appearances of the word $w_{i}$, $\left |{ D }\right |$ stands for the number of documents and ${\mathrm {DF}}_{(w_{i})}$ is the number of documents containing the term $w_{i}$. The TF-IDF statistical measure is used throughout the present study for features representation.

By only focusing on the number of times a word occurs in a given text, the Bag-of-Words approach does not provide any information regarding the succession of the words. This issue can be addressed if the n-gram language model is used, in which the text is represented through successions of $N$ consecutive words. Common types of n-grams include grams of size one, called unigrams (1-grams), grams of size two, called bigrams (2-grams), and grams of size three, called trigrams (3-grams) [94].

In the present study, various combinations of unigrams, bigrams and trigrams have been considered as features for the machine learning algorithms, as shown in Table 5.

**TABLE 5:** N-Gram Combinations

N-gram model	Types of n-grams
(1-1)	unigrams
(1-2)	unigrams + bigrams
(1-3)	unigrams + bigrams + trigrams
(2-2)	bigrams
(2-3)	bigrams + trigrams
(3-3)	trigrams

Besides the Bag-of-Words representation, word embeddings have been used. In word embeddings the words are mapped to vectors, having similar representations for the words which frequently appear in the same context. Compared to one-hot encodings, word embeddings provide a denser representation that requires a smaller number of dimensions for representing the words. The similar representation of words with close meanings provides additional clues for the classification algorithms. The following word embeddings have been considered in the present study: Datastories,^3^ GloVe^4^ and Fast-Text.^5^^3^https://github.com/cbaziotis/datastories-semeval2017-task4^5^https://nlp.stanford.edu/projects/glove^5^https://fasttext.cc/docs/en/english-vectors.html

### C. Learning Algorithms

A machine learning approach has been used in order to accurately determine the stance towards vaccination in the collected tweets. Starting from the annotated dataset, the performance of several popular classification algorithms has been investigated: Multinomial Naive Bayes (MNB), Random Forest (RF), Support Vector Machine (SVM), Bidirectional Long Short-Term Memory (Bi-LSTM) and Convolutional Neural Network (CNN).

#### 1) Multinomial Naive Bayes

Naive Bayes classifiers are a family of probabilistic classification algorithms that apply the Bayes theorem. They are called naïve because they perform the classification under a strong assumption that every feature is independent from the other features. Despite their simplicity, this family of algorithms has been demonstrated to be fast, reliable and accurate in many NLP classification tasks [95]. The Multinomial Naive Bayes [96] classifier implements a variant of the Naïve Bayes algorithm which can be used with multinomially distributed data, such as the frequencies of n-grams in text classification problems.

#### 2) Random Forest

Random Forest (RF) [97] is an ensemble classifier that consists of multiple decision tree classifiers, trained in parallel with bootstrapping followed by bagging. According to Misra and Li [98] the RF classifier offers better results when compared to other classification methods in terms of accuracy and does not require feature scaling. Furthermore, the RF classifier has been determined to be more robust in the selection of training samples. Even though the RF might be hard to interpret, its hyperparameters can more easily be turned than in the case in which a decision tree classifier is used [98].

#### 3) Support Vector Machine

Support Vector Machines (SVM) [99] are a family of supervised learning algorithms used for classification, regression and other tasks such as outlier detection. While other classification algorithms suffer from overfitting, one of the advantages of SVM is that they are less prone to this situation [100]. Another advantage resides in the fact that besides binary classification, multiclass classification can be performed by combining several binary classification functions. For this, each class is considered individually at a time, and for each class a classifier is searched that separates it from the other classes [101].

#### 4) Long Short-Term Memory

The Long Short-Term Memory (LSTM) [102] is a type of Recurrent Neural Network (RNN). In the current paper, a bidirectional LSTM approach has been used (Bi-LSTM), that follows the architecture proposed by Baziotis *et al.* [92], which has ranked among the best two submissions at “SemEval-2017 Task 4” [103]. The architecture consists of the following layers: word embedding (none, 50, 300), Gaussian noise (none, 50, 300), bidirectional LSTM (none, 50, 300), bidirectional LSTM (none, 50, 300), attention (none, 300), dropout (none, 300), dense (none, 3) and activation (none, 3). The Gaussian noise and bidirectional LSTM layers are followed by dropout (none, 50, 300) layers.

#### 5) Convolutional Neural Network

Convolutional Neural Networks (CNN) are a type of neural networks that are specialized for processing data that features a grid-like topology [46]. CNNs have already been successfully used in different NLP tasks, including stance classification [5], [104], [105].

In the current paper, we have followed the approach used by Cliché [104] and Baziotis *et al.* [106] regarding the filter lengths of [3]–[5]. Additionally, the architecture of the network is similar to the one presented in [106]. In the approach used in the current paper, the word embedding layer (none, 50, 300) is followed by a Gaussian noise layer (none, 50, 300) and by a dropout layer (50, 300). After this layer, three 1-D convolutional layers (using ReLU activation) have been added, each followed by a max pooling layer and a flattening layer. The outputs of these layers are merged in a concatenation layer (none, 7000). The dropout layer (none, 7000) and a dense layer (none, 3) conclude the network.

#### 6) Bidirectional Encoder Representations From Transformers

Bidirectional Encoder Representations from Transformers (BERT) [63] is a pre-trained transformer-based language model. Compared to word embeddings such as GloVe, BERT has the advantage of taking into account the context for each occurrence of a given word. The model has been pre-trained on a diverse corpus of unlabeled text extracted from the English Wikipedia and the BookCorpus [107].

Pre-trained BERT models with a wide range of sizes exist, varying the number of layers $L$ from 2 to 24 and the hidden size $H$ from 128 to 1024 [63], [108]. In the present paper, the BERT_BASE_^6^ model has been chosen, having $L=12$, $H=768$ and the number of self-attention heads $A=12$. The neural network architecture has a total of 110M parameters. In comparison, BERT_LARGE_ ($L=24$, $H=1024$, $A=16)$, having 340M parameters, has been shown to provide improvements in accuracy of no more than 5% [63], while being far more compute intensive.^6^https://huggingface.co/bert-base-uncased

### D. Experiments and Results

The classical machine learning algorithms have been implemented using the scikit-learn [109] library, while the deep learning algorithms have been implemented using the Keras^7^ library, having TensorFlow^8^ as a backend.^7^https://keras.io^8^https://www.tensorflow.org

Cross-validation using either 5-folds [51] or 10-folds [5] is a widely-used approach for comparing and selecting classifiers. In this paper, following the approach described by Mohammad *et al.* [51], the classifiers have been evaluated through a 5-fold cross-validation procedure, during which the classification model is trained using k-1 of the folds as training data, while the resulting model is validated on the remaining part of the data. The performance of the classifier is then computed as an average of the values computed during the k consecutive runs. Since the balanced dataset includes 3249 tweets (1083 in each class), at each iteration, the classification models are trained using 2600 tweets and evaluated using the remaining 649 tweets. The results of the considered methods are shown in Table 6 and further discussed in the sub-sections below.

**TABLE 6:** Classification Performance

Code	Classifier	Class	Precision	Recall	F-score	Accuracy
C1	MNB n-gram: (1, 2); features: 3000	against	70.57%	83.74%	76.59	73.71%
		neutral	79.80%	72.20%	75.78	
		in favor	71.77%	65.20%	68.31	
C2	MNB n-gram: (1, 2); features: 2000	against	70.86%	83.37%	76.58	73.34%
		neutral	78.34%	73.50%	75.82	
		in favor	71.51%	63.16%	67.04	
C3	RF n-gram: (1, 1); features: all	against	67.99%	83.10%	74.77	71.71%
		neutral	76.19%	73.13%	74.60	
		in favor	72.20%	58.91%	64.84	
C4	RF n-gram: (1, 2); features: 2000	against	69.39%	81.25%	74.84	70.79%
		neutral	71.50%	74.70%	73.04	
		in favor	72.12%	56.42%	73.04	
C5	SVM n-gram: (1, 2); features: all	against	74.43%	84.03%	78.93	76.23%
		neutral	80.50%	76.18%	78.25	
		in favor	74.21%	68.52%	71.20	
C6	SVM n-gram: (1, 2); features: 2000	against	73.09%	78.76%	75.80	74.20%
		neutral	76.84%	77.84%	77.32	
		in favor	72.74%	66.03%	69.16	
C7	SVM word embedding: glove.6B.100d	against	70.79%	73.70%	71.45	68.88%
		neutral	72.39%	74.51%	73.14	
		in favor	66.44%	58.46%	60.95	
C8	Bi-LSTM word embedding: datastories.twitter.300d	against	74.62%	72.83%	73.65	73.41%
		neutral	78.74%	76.38%	77.48	
		in favor	67.66%	71.10%	69.20	
C9	Bi-LSTM word embedding: glove.twitter.27B.200d	against	74.85%	77.80%	76.04	74.70%
		neutral	79.75%	76.26%	77.79	
		in favor	70.30%	70.19%	70.17	
C10	Bi-LSTM word embedding: fasttext.wiki-news-300d	against	60.43%	87.59%	71.24	68.36%
		neutral	79.02%	70.94%	74.55	
		in favor	72.48%	47.16%	56.91	
C11	CNN word embedding: datastories.twitter.300d	against	70.50%	60.48%	64.65	65.71%
		neutral	68.97%	75.38%	72.02	
		in favor	58.94%	61.57%	59.95	
C12	CNN word embedding: glove.twitter.27B.200d	against	71.19%	66.13%	68.26	68.91%
		neutral	71.04%	77.80%	74.21	
		in favor	65.31%	62.65%	63.68	
C13	CNN word embedding: fasttext.wiki-news-300d	against	67.72%	71.17%	69.26	69.01%
		neutral	72.17%	77.50%	74.66	
		in favor	66.63%	58.20%	62.07	
C14	BERT	against	74.55%	84.52%	79.20	78.94%
		neutral	86.27%	79.07%	82.34	
		in favor	77.53%	73.29%	75.28	

#### 1) BoW and Classical Machine Learning

The best parameters for the developed natural language processing pipeline have been determined through the grid search approach. Thus, different numbers of features have been tested, including using all the features and reducing the number of features, $F$, to a maximum of 1500, 2000 and 3000 values.

Different n-gram combinations, ranging from (1,1) to (1,3), as listed in Table 5, have been investigated for the string vectorizer, as well. Additionally, the algorithms have been evaluated considering both the case in which the general stop words are kept and the one in which they are excluded. The stop word list that has been considered is the one included in the NLTK library. In the case of the corpus-specific stop words, the document frequency thresholds, maxDF, that have been considered are 0.5, 0.75 and 1.0. Besides, the evaluation has also analyzed whether applying Term Frequency (TF) or Term Frequency - Inverse Document Frequency (TF-IDF) can improve the stance classification results.

We have experimented with different settings for the classifiers, including varying, in the case of the SGDClassifier, the alpha parameter, which multiplies the regularization term. Different regularization terms have been tested, including “l1”, “l2” and “elasticnet”. The loss function of the SGDClassifier has been configured as “hinge”, corresponding to a linear SVM.

For each considered classical machine learning classifier (C1–C6), Table 6 includes both the results achieved using the parameters determined through grid search (C1, C3, C5) and the results achieved through the n-gram model (1, 2), corresponding to unigrams and bigrams, and limiting the maximum number of features to $F=2000$. For the C1–C6 classifiers, the best results have been achieved when using TF-IDF, without excluding the general stop words.

In the case of the Multinomial Naïve Bayes classifier (C1 and C2) the best results have been achieved for the C1 classifier, for which the maximum number of features has been reduced to $F=3000$, while including both unigrams and bigrams as features, keeping maxDF = 1.0.

It has been observed that the Random Forest classifier (C3 and C4) performed best when using only unigrams, without limiting the number of features, while applying a frequency threshold, maxDF, for corpus specific stop words of 0.5, namely in the case of C3.

In the case of the Support Vector Machines classifier (C5 and C6), the best results have been achieved in the case of C5, configured with the n-gram model (1, 2), without limiting the maximum number of features, an alpha parameter value of 0.0001, a maxDF threshold equal to 1.0 and choosing “elasticnet” as a regularization term.

As expected, C1, C3 and C5, for which the parameters have been determined through grid search have performed better than the corresponding classifiers of the same type, C2, C4 and C6.

The overall best performing classifier has been C5, a SVM classifier which had 76.23% accuracy, followed by C6, with 74.20% accuracy. In terms of precision and F-score, C5 overperformed all the other classifiers for each of the three considered classes, *in favor*, *against* and *neutral*. A small difference is recorded in the case of recall, where the value for the *neutral* class is slightly lower for C5 than for C6 (76.18% versus 77.84%).

The worst performing classifier has been C4, a RF classifier, with an accuracy of 70.79%. In terms of precision and recall, the classifier C4 performed worse than C5 and C6 on all three classes, *in favor*, *against* and *neutral*.

#### 2) Word Embedings and Classical Machine Learning

Starting from the algorithm that has provided the best results in the context of the Bag-of-Words approach, C5, in the following we have analyzed if the performance can be further improved by considering pre-trained word embeddings. Similar approaches, using word embeddings with classical machine learning algorithms, have been investigated in [5] and [110].

To this end, a word embedding, called glove.6B, that includes six billion tokens, created through the Glove approach from a corpus extracted from Wikipedia and from the news archive Gigaword [111], has been used. This implementation is marked in Table 6, as C7.

As shown in Table 6, the values of all the four considered metrics (precision, recall, F-score and accuracy) of the C7 classifier are worse than those achieved in the case of C5.

#### 3) Word Embedings and Deep Learning

In the case of the deep learning classifiers, in the present paper, the Adam approach has been applied for tuning the learning rate [112]. The resulting classifiers are listed in Table 6 under the C8 – C13 classifiers.

As shown in Table 6, among the Bi-LSTM classifiers (C8–C10), the best results have been achieved by the C9 classifier, with an accuracy of 74.70%, higher than in the case of C8, (73.41%) and C10 (68.36%). The C9 classifier has used the word embeddings created through the Glove approach from a corpus composed of 2 billion tweets.

In the case of CNN classfieirs (C11–C13) the best results have been achieved by the C13 classifier, using the word embeding created through the FastText approach on a corpus extracted from Wikipedia and news stories (accuracy 69.01%).

Classifiers C8 and C11, ranked second in the Bi-LSTM category (73.41%) and third in the CNN category (65.71%) based on accuracy, have used the Datastories word embeddings, created from a corpus of 330 million tweets, by applying the GloVe approach.

The best performing deep learning classifier has been C9, implementing Bi-LSTM, which outperforms C8, C10–C13 classifiers, both in terms of accuracy and F-score.

#### 4) Bidirectional Encoder Representations From Transformers

In order to establish the best values for the hyperparameters of the BERT language model (C14), the approach recommended by Devlin *et al.* [63] has been followed during the fine-tuning procedure in regarding the batch sizes (16, 32), learning rate (5e-5, 3e-5, 2e-5) and number or epochs (2, 3, 4). The best results have been achieved when using a batch size of 16, a learning rate of 3e-5 and a number of epochs equal to 3. Having an accuracy of 78.94%, the C14 classifier outperforms all the other classifiers. Moreover, it clearly outperforms the second-best performing classifier, C5, in terms of precision, recall and F-score, for all the considered classes.

### E. Discussion

The results achieved by the deep learning classifier C9 are worse than the ones obtained in the case of the classical machine learning classifier C5 in terms of accuracy and F-score. This result is consistent with the ones in other studies, such as D’Andrea *et al.* [5], in which classical machine learning algorithms have outperformed deep learning approaches, such as CNN and LSTM, in the case of vaccine stance classification.

As noted in the review paper of Wang *et al.* [31], stance detection approaches typically do not perform extremely well. The reasons mentioned by the authors include the sparsity, the colloquial language and the absence of large, labeled datasets that could be used for training. Moreover, Mohammad *et al.* [51] summarize the results of the “SemEval-2016 Task 6: Detecting Stance in Tweets” mentioning that the SVM baseline with n-grams has performed relatively well compared to other machine learning approaches.

In the following we have used the best performing classifier, C14, to analyze the tweets collected over the considered period of time. The model has been trained on all the tweets in the annotated dataset.

## VI. Analyzing Social Media

The evolution of the daily number of tweets is discussed in this section in connection with the major events which have occurred around the world related to COVID-19 vaccination, with an accent on the English-speaking countries.

### A. Major Events

The major events have been extracted from the news published online in each day of the analyzed period using google.com search engine by selecting the “News” section and “COVID” keyword and by pointing one-by-one the days in the mentioned period. Each time, the first 10 pages of News titles have been considered and the most relevant news have been extracted in connection to the COVID-19 vaccination theme, relevance being given by the connection to the COVID-19 vaccination and the amount of news on a specific topic.

As a result, it has been observed that in all the analyzed days there have been news regarding the COVID-19 vaccination theme, starting from the announcement of the vaccine effectiveness by different producers, the amount of money funded by various organizations for COVD-19 vaccine, adverse events encountered in the pre-test phase, ethical issues related to whom should have first access to the vaccine, the predicted quantity of vaccines to be distributed in different countries and areas and ending with the vaccination in Russia and UK.

The following events have been put in connection with the number of tweets recorded daily, which might have determined the variation in the tweets’ number:

E1. Nov. 9: Pfizer and BioNTech announcement regarding their COVID-19 vaccine effectiveness^9^
E2. Nov. 10: Positive news regarding stock trading, oil futures and cruise bookings rise as a result of COVID-19 vaccine^10^^,^^11^
E3. Nov. 13: World Health Organization exceeded the target of $ 2 billion to buy and distribute COVID-19 cures to poorer countries^12^
E4. Nov. 16: Moderna’s COVID-19 vaccine shows 94.5% efficiency in clinical trials^13^
E5. Nov. 18: Sinovac’s COVID-19 vaccine induces a quick immune response^14^
E6. Nov. 20: Pfizer’s announcement regarding COVID-19 vaccine emergency authorization^15^
E7. Nov. 23: Oxford AstraZeneca COVID-19 vaccine shows an up to 90% efficacy^16^
E8. Nov. 27: UK hospitals start preparing for the arrival of the COVID-19 vaccine in 10-day time^17^
E9. Nov. 30: Moderna seeks approval for the COVID-19 vaccine in Europe and United States^18^
E10. Dec. 2: UK authorize the Pfizer BioNTech COVID-19 vaccine^19^
E11. Dec. 3: The first batch of vaccines arrived in UK^20^
E12. Dec. 8: UK starts COVID-19 vaccination^21^

^9^https://www.cnbc.com/2020/11/09/covid-vaccine-pfizer-drug-is-more-than-90percent-effective-in-preventing-infection.html (accessed December 9, 2020)^10^https://www.cnbc.com/2020/11/10/cruise-bookings-rise-on-coronavirus-vaccine-news-norwegian-cruise-line-ceo-says.html (accessed December 9, 2020)^11^https://in.reuters.com/article/global-oil/oil-gains-after-stockpile-draw-amid-hopes-for-coronavirus-vaccine-idINL4N2HX0O6 (accessed December 9, 2020)^12^https://uk.reuters.com/article/uk-health-coronavirus-vaccines-covax-idUKKBN27T138 (accessed December 9, 2020)^13^https://www.ft.com/content/9d7a2e24-aea0-4c45-82ab-509dc80ed5a1 (accessed December 9, 2020)^14^https://www.reuters.com/article/uk-health-coronavirus-sinovac/sinovacs-covid-19-vaccine-induces-quick-immune-response-study-idUKKBN27 × 35I (accessed December 9, 2020)^15^https://www.technologyreview.com/2020/11/20/1012391/pfizer-authorization-covid-19-vaccine-christmas/ (accessed December 9, 2020)^16^https://www.theguardian.com/society/2020/nov/23/astrazeneca-says-its-coronavirus-vaccine-has-70-per-cent-efficacy-covid-oxford-university (accessed December 9, 2020)^17^https://www.theguardian.com/world/2020/nov/27/hospitals-england-told-prepare-early-december-covid-vaccine-rollout-nhs (accessed December 9, 2020)^18^https://www.bbc.com/news/health-55129336 (accessed December 9, 2020)^19^https://www.economist.com/science-and-technology/2020/12/01/britain-becomes-the-first-country-to-license-a-fully-tested-covid-19-vaccine (accessed December 9, 2020)^20^https://www.bbc.com/news/uk-55181665 (accessed December 9, 2020)^21^https://www.bbc.com/news/uk-55227325 (accessed December 9, 2020)

In order to validate the correspondence between the events and the analyzed tweets we have extracted for each date in the analyzed period the unigrams, bigrams and trigrams sorted according to the number of appearances. The analysis has been performed for both the cleaned dataset and the whole dataset, that also includes the retweets. Before the n-gram extraction, the tweets have been minimally pre-processed by removing stop words and duplicated white spaces.

From the events presented above, we have selected two events, one that has generated a large number of tweets, namely E10, and another one that has generated a comparatively smaller number of tweets, namely E6.

Analyzing the n-grams for the 154?004 tweets collected for December 2, the day of E10, it has been observed that among the top-15 unigrams, besides the specific COVID-19 terms (e.g. “vaccine”, “covid”, “19”, “coronavirus”, “covid19”, “vaccines”) in this day “Pfizer” has been referred 57?342 times, followed by “UK” referred 48 789 times, “first” referred 39 438 times, “BioNTech” referred 30 993 times and “approve” referred 18 949 times. Based on the top-10 bigrams and trigrams, it can be observed the occurrence of the following words’ combinations: “Pfizer BioNTech” referred 29 714 times, “first country” referred 18 085 times, “approve Pfizer” referred 15 453 times. Considering the extracted unigrams, bigrams and trigrams and the E10 event, UK authorization of the Pfizer and BioNTech COVID-19 vaccine, it can easily be noted that there exists a correspondence between E10 and the analyzed tweets from December 2.

On November 20, there have been collected 59 674 tweets. From the top-15 unigram analysis, the following have been extracted: “Pfizer” (17 639 times), “emergency” (13 466 times), “authorization” (7251 times), “fda” (6622 times) and “BioNTech” (5347 times). As for the top-10 bigrams and trigrams, the words’ combinations have been: “emergency use” (9613 times), “use authorization” (4769 times), “emergency use authorization” (4768 times) and “Pfizer BioNTech” (4583 times). It can thus be observed that even in the case of a less significant event (“Pfizer’s announcement regarding COVID-19 vaccine emergency authorization”) the correspondence between the tweets and the event exists.

### B. Stance Analysis

In the following, the best performing classifier - determined in Section V, BERT (C14) - is used to perform stance analysis on the gathered dataset. As it will be observed, not all the news published in the analyzed period have generated the same amount of interest from the general public, a series of local peaks being identified in some of the analyzed days.

#### 1) Cleaned Tweets Stance Analysis

**FIGURE 2.**
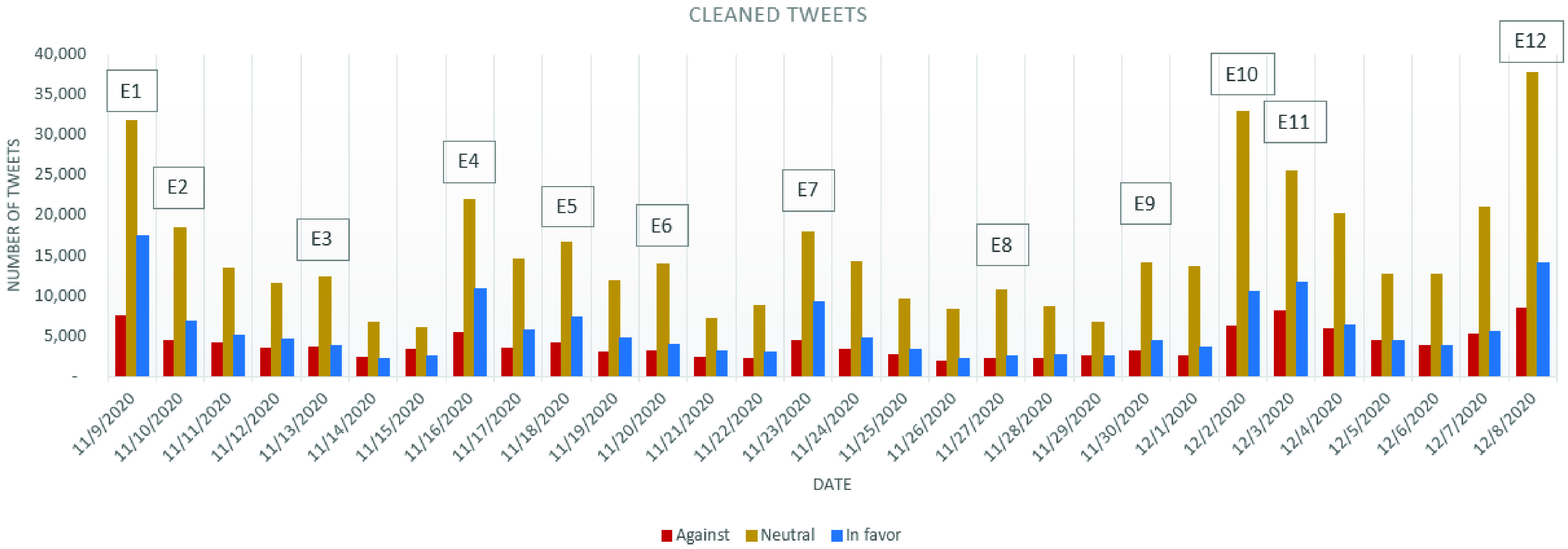
Evolution of the cleaned tweets stance.

The evolution of the stance expressed and the distribution of the stances on the three considered categories: *in favor*, *against* and *neutral* is depicted in Figure 2, which considers only the cleaned tweets. By simply considering the stances’ evolution, one can easily observe that there have been variations in the number of tweets published, especially in the days following a major announcement or news.

From Figure 2 it can be observed that the general dominating stance is *neutral*, as it was expected from the initial stage in which most of the annotated tweets belonged to this category. Based on the tweets in the *neutral* category, it has been noticed that most of them deal with presenting news related to the occurrence of the COVID-19 vaccine.

As for the number of *against* and *in favor* tweets, it can be observed that they have oscillated during the analyzed period. Based on Figure 2 it can be observed that between November 9 and December 1 the number of *against* tweets has kept a constant trend, with a few “spikes” on November 9, November 16 and November 23. In all of these days, the news released in the media were speaking about the efficiency of COVID-19 vaccines in clinical trials: for Pfizer and BioNTech on November 9 (event E1), for Moderna on November 16 (E4) and for Oxford AstraZeneca on November 23 (E7). Two major turning points for increasement in the number of *against* tweets have been represented by December 2 and December 8, these being the days in which UK has authorized the Pfizer BioNTech COVID-19 vaccine (E10, 6242 *against* tweets) and the day in which the COVID-19 vaccination started in the UK (E12, 8429 *against* tweets).

Considering the evolution of the graphic containing the cleaned tweets from all the three categories, it can be observed that the number of *against* tweets follows on a smaller scale the evolution of the number of *neutral* tweets (the curve of the *against* tweets being more flattened than in the case of the *neutral* tweets), while the *in favor* tweets follow more precisely the trend imposed by the number of *neutral* tweets (Figure 2; Figure 7 and Figure 8). As a result, for the *in favor* tweets one can observe an increasement in most of the major events cases presented above. Even more, in the events announcing the effectiveness of the COVID-19 vaccines from different companies (E1, E4, E5 and E7) the *in favor* tweets have overpassed the number of *against* tweets. Even after the vaccine authorization in UK, the situation has not changed and the number of *in favor* tweets overpassed daily the number of *against* tweets (with approximately, on average, 2050 tweets per day).

**FIGURE 3.**
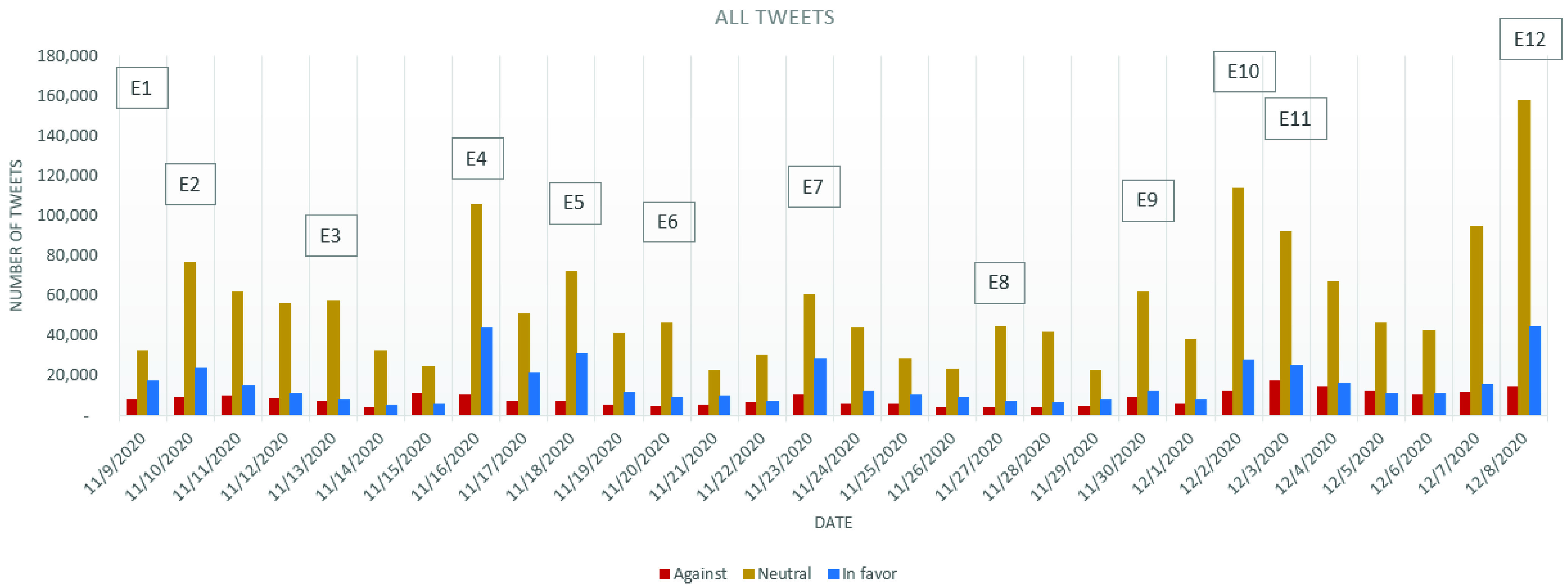
Evolution of all tweets stance.

**FIGURE 4.**
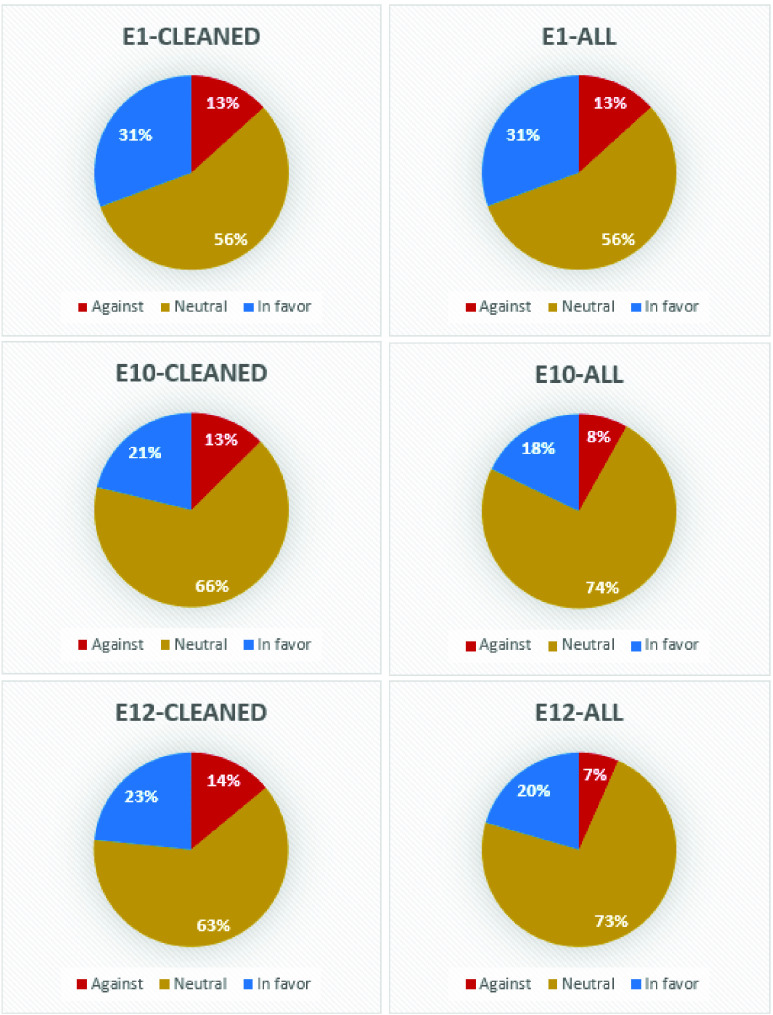
Context related event comparison (for E1, E10, E12).

**FIGURE 5.**
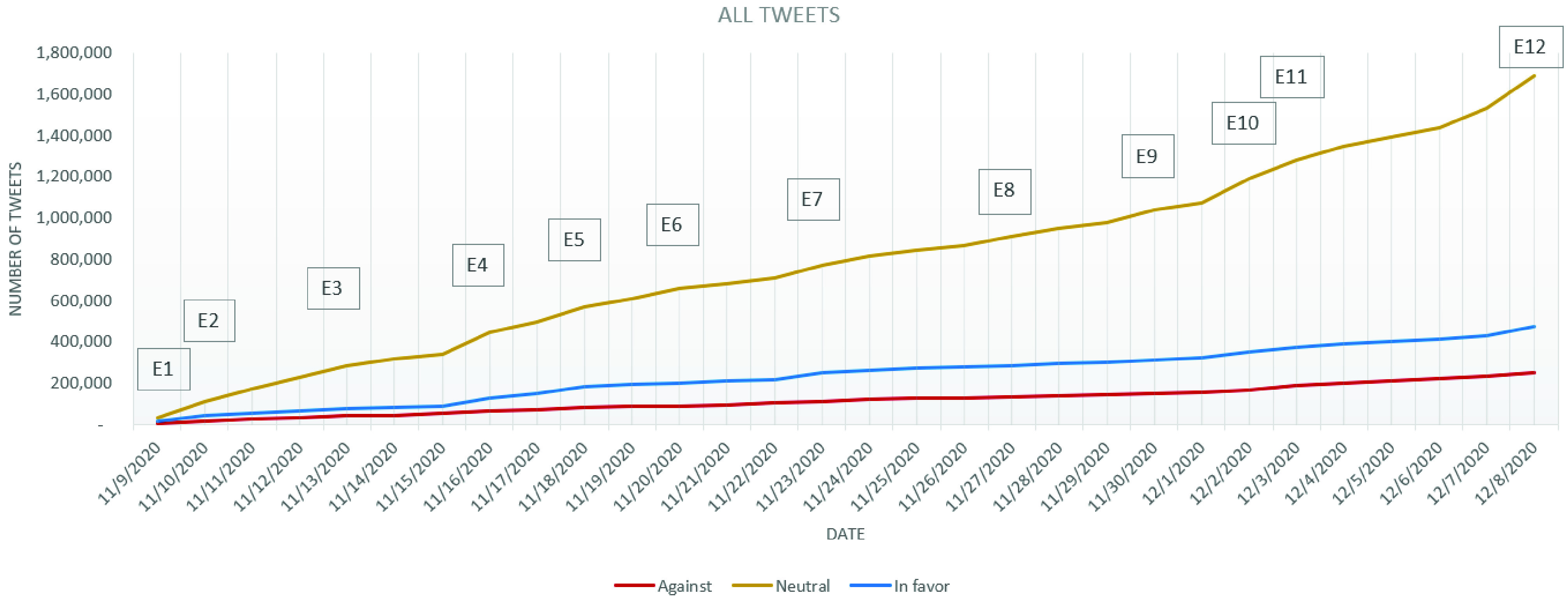
Cumulative stance analysis evolution.

**FIGURE 6.**
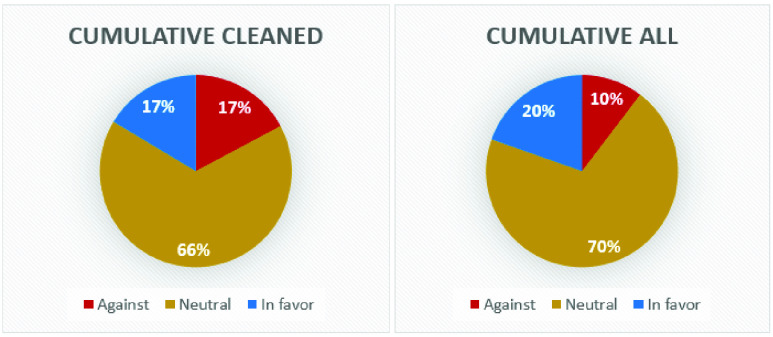
Cumulative stance comparison.

**FIGURE 7.**
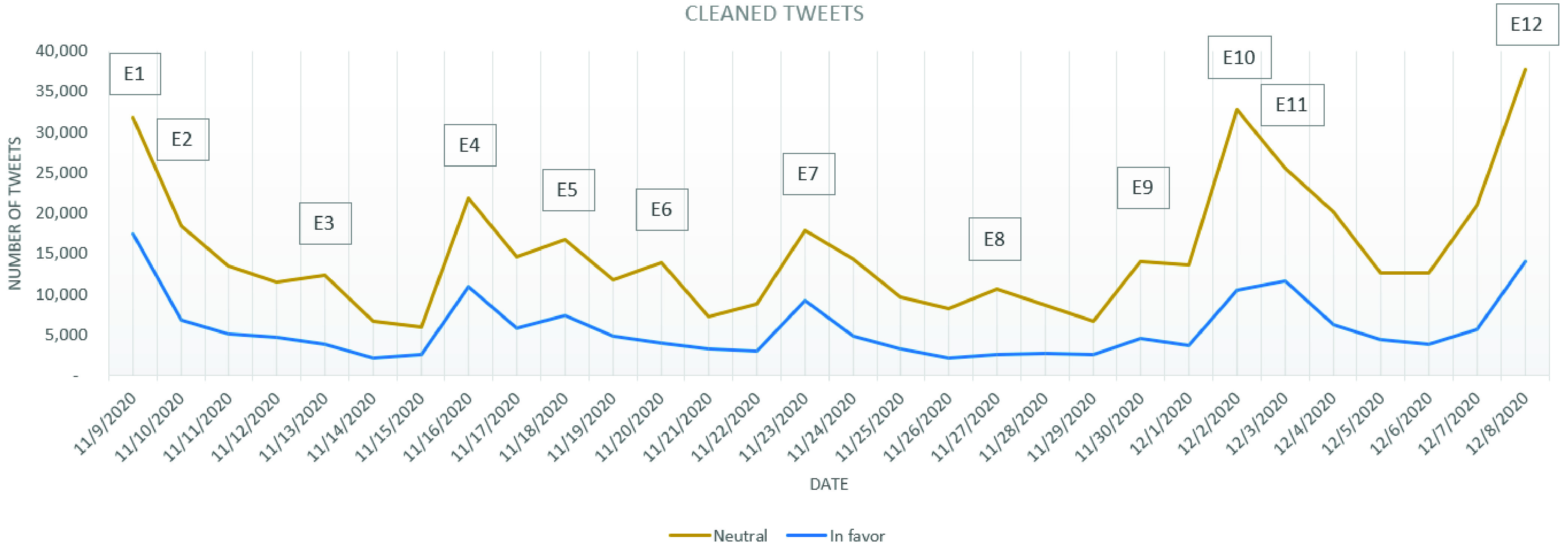
Evolution of *neutral* and in *favor* cleaned tweets.

**FIGURE 8.**
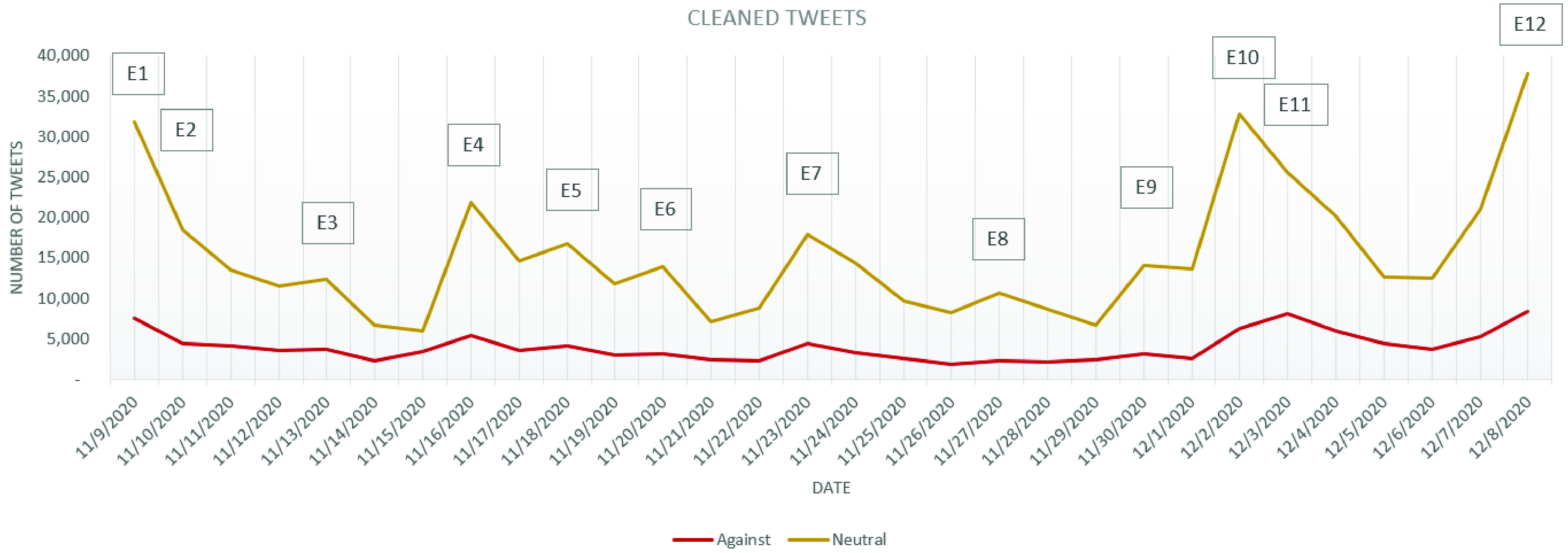
Evolution of *neutral* and *against* cleaned tweets.

#### 2) All Tweets Stance Analysis

As the difference, in the number of tweets, between the *in favor* and *against* stances in the cleaned dataset seem to have close values during the analyzed period, a stance analysis considering all tweets dataset has been performed.

In the study conducted on vaccination in Italian tweets, D’Andrea *et al.* [5] considered that one should analyze the whole tweets dataset (including retweets) as, some of the users who retweet a certain opinion or piece of information, generally believe in it and, instead of writing their own words to a particular situation, they might decide instead to share the information. As a result, we have run the stance analysis over the entire tweet-dataset and the results are presented in Figure 3.

As it can be observed from Figure 3, on November 9, when Pfizer and BioNTech announced their vaccine effectiveness, the number of retweets has been only slightly different from the ones of tweets (a difference of 497 tweets). On November 10, the number of cleaned tweets has been half compared to November 9, while the total number of tweets doubled, showing a high increasement in the number of retweets. As the events marking November 10 were mostly referring to the economic impact of a possible COVID-19 vaccine, such as the rose of oil futures and the announcement of a growth in stock market, in general, an increasement in the *in favor* tweets over the *against* tweets can be observed (which has not been observed in the cleaned tweets dataset).

**FIGURE 9.**
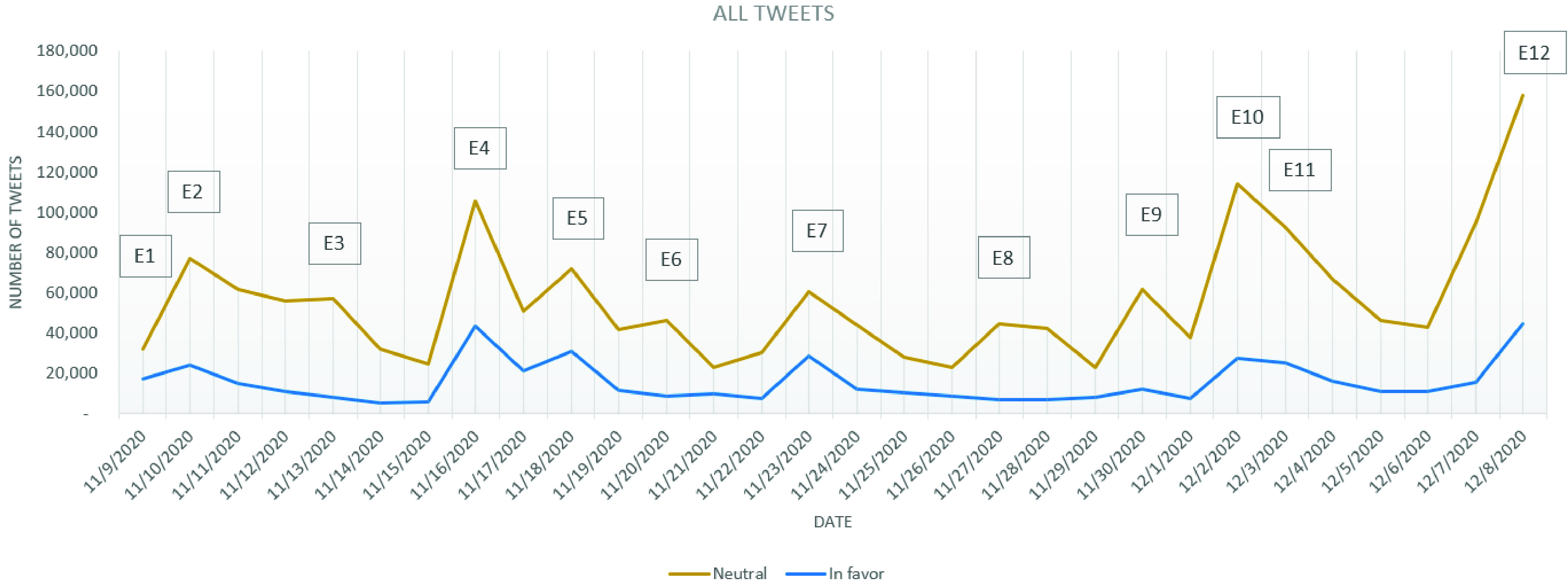
Evolution of *neutral* and in *favor* for all tweets.

**FIGURE 10.**
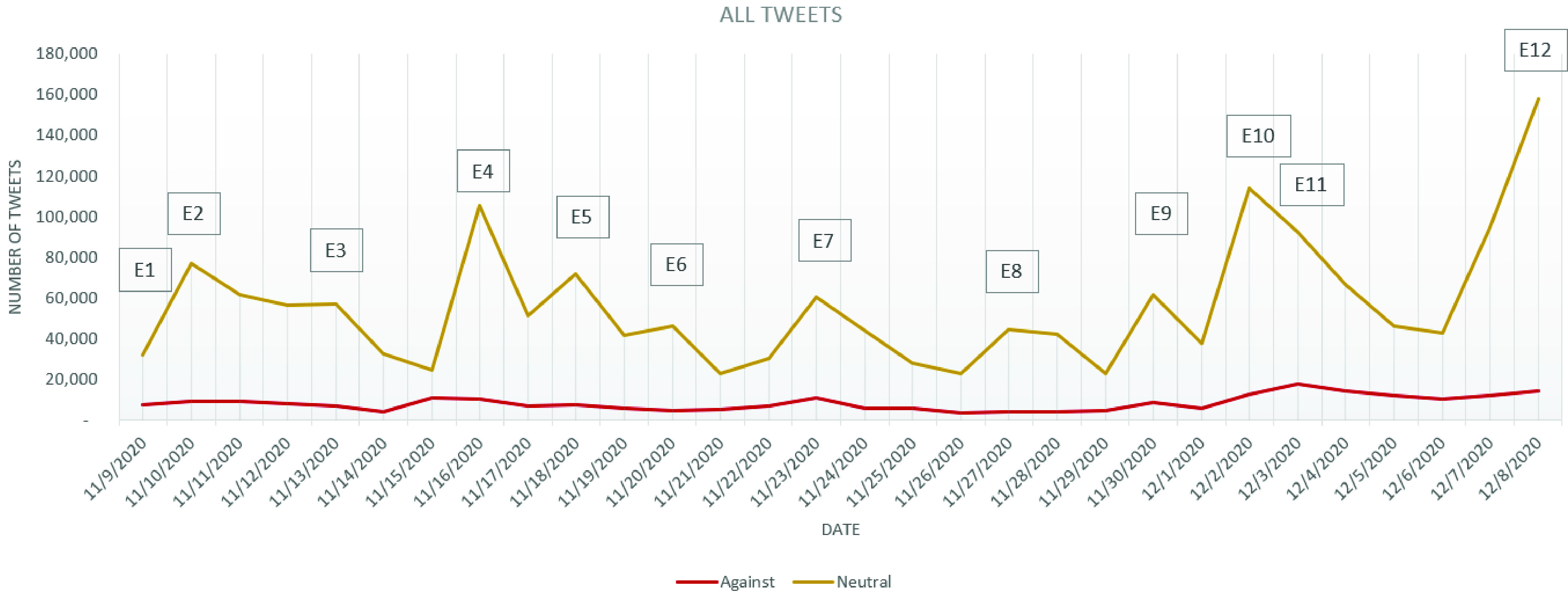
Evolution of *neutral* and *against* for all tweets.

Even in this case, one can notice that the evolution of the number of *in favor* tweets resembles more closely to the evolution of the *neutral* tweets. The increasement in the number of tweets marked as *in favor* is better visible in the case of the occurrence of the events mentioned above E1–E12, more precisely in the cases of E2, E4, E5, E7, E10 and E12 (Figure 9), than in the other days of the analyzed period. For the *against* tweets, except for the increasement observed in the period following E10 (Figure 10), the evolution is almost constant, recording an average number of *against* tweets of approximately 6826 tweets/day. After E10, the number of daily *against* tweets recorded an increasement of 95.28%.

Comparing the number of tweets recorded in the three categories for the period between December 2 – December 8 (E10–E12) with the period between November 9 – December 1 (E1–E9), it can be observed that the higher relatively increasement has been recorded in the *against* tweets category (95.28%), followed by *neutral* (87.86%) and *in favor* (54.86%). The smaller difference noticed between the number of tweets in the *against* category compared to the *in favor* category can be attributed to the fact that starting from December 2 (E10) the potential of having a vaccine was no longer a “dream” but it became “reality”, which might have “activated” the anti-vaccination community.

#### 3) Context Related Event Comparison

For better understanding the opinion polarity in connection with a given event, we have considered in the following, three major events recorded in this month: first announcement of a possible effective vaccine (E1), first authorization of a vaccine (E10) and first vaccination (E12), all of them creating spikes in the number of tweets.

As a result, it has been decided to aggregate the opinions expressed in the days corresponding to the three events for both the cleaned and for the entire data set. The results have been summarized in Figure 4.

A first observation is related to the fact that the stance is overall *neutral* for the considered events. Particularly, in terms of relative distribution, one can note that that percentage of the *neutral* tweets in total number of tweets has recorded a slight increase of 17% (from 56% for E1 to 73% for E12) when all the tweets are considered and an increase of 7% on cleaned tweets (from 56% for E1 to 63% for E12) – please see Figure 4.

A higher percentage variation, in relative terms, can be observed for the *in favor* tweets: on the entire dataset the percentage dropped from 31% to 20%, while in absolute value, the number of *in favor* tweets almost doubled from 17 526 tweets for E1 to 44 447 tweets for E12 (with 27 487 tweets for E10).

A decrease in the percentage of tweets from the entire sample can be also observed in the case of *against* tweets, even though, in this case, the decrease is of only 6% (from 13% to 7%). Considering the cleaned tweets, the percentage of the *against* tweets has faced an increase of 1% (between E1 and E10–E12). Even in this case, the absolute number of *against* tweets on all dataset increased from 7604 tweets on E1 to 12 541 on E10 and to 14 359 on E12.

Another observation can be made in connection to E1 and E10: it can be observed that in the case of the *against* tweets, the increasement of the number of these tweets happened mostly in the following days after the events occurred, not being so visible in the day of the event. This observation is in line with the study conducted by D’Andrea *et al.* [5], in which the authors have shown that sometimes the effect of an event is immediately visible, while in some other cases, it might need some hours / a day in order to be visible in the tweets. A possible explanation for this lag in response might be related to the fact that the users posting *against* tweets might have needed some time in order to look for information related to the event prior to tweeting.

#### 4) Cumulative Stance Analysis

As expected, the predominant stance of the cumulative set is *neutral* both in the entire set (Figure 5) and in the cleaned set.

The percentage of the *against* and *in favor* reaches the same value in the case of cleaned tweets, even though in absolute value, there can be noticed a difference in favor of the *against* tweets (Figure 6). On the entire dataset, the *in favor* tweets have a 10% difference compared to the *against* tweets (Figure 6). As the difference between the *in favor* and *against* tweets is not as visible as expected (Figure 6) and a global vaccination campaign is expected, the involved agencies and governments should try to provide more information regarding the vaccination process, its advantages and its presumed disadvantages, offering to the general public all the needed instruments and information for increasing their trust in the decisions taken at a macro-scale, with impact on everybody’s life.

## VII. Limitations of the Study

A potential limitation of the current study is represented by the classification algorithms selected in the paper. As the Natural Language Processing field is in continuous development, better algorithms could be developed over time providing an improved classification performance. Another limitation is related to the selected dataset, which only includes tweets extracted between November 9, 2020 and December 8, 2020, written in English. This limitation opens the path towards other possible extensions, which might include, but not limited to analyzing the tweets written in other languages or from specific geographical areas, which could be identified using the associated GPS coordinates. Additionally, a different period of time could be considered due to the vaccination process dynamics worldwide.

## VIII. Conclusion

In the current paper, the one-month period passed between the first announcement of a coronavirus vaccine and the first actual vaccination process started outside the limited clinical trials has been analyzed using machine learning-based stance detection. Multiple classical machine learning and deep learning algorithms have been compared and the best performing classifier has been chosen based on four performance metrics.

The proposed approach has classified the tweets into three main classes, namely *in favor*, *against* and *neutral* regarding COVID-19 vaccination, employing BERT with an accuracy of 78.94%.

The aim of the paper has been to monitor the evolution of the stance towards COVID-19 vaccination from tweets, by matching the number of Twitter messages with the main events reported by the media in the analyzed period.

The main stance was *neutral* at both a daily level and for the entire period, on either the cleaned dataset or on the complete dataset (dataset including all the tweets). In terms of *neutral* tweets’ percentage recorded at the beginning of the period, November 9, versus the end of the analyzed period, December 8, it has been observed that the percentage has increased with 17% on the complete dataset. The evolution of the *in favor* tweets has been characterized by a series of spikes, closely following the evolution of the *neutral* tweets, but at a reduced scale. For both the *neutral* and *in favor* tweets it has been observed that some of the events in media have entrained a series of spikes, which are not encountered in the case of *against* tweets, where the major spike has been represented by the authorization in the UK of the Pfizer BioNTech COVID-19 vaccine.

Even more, it has been observed that in the case of the *against* tweets there is sometimes a lag between the day in which a certain event has occurred and the change in the number of *against* tweets. The existence of such a lag in tweets posting has also been mentioned by D’Andrea *et al.* [5] in a paper regarding vaccination in Italy.

The correspondence between the information in the tweets and the events in the media has been confirmed by analyzing the n-grams, underlining even more the fact that the tweets reflect the hot topics in the society at large.

The early detection of an opinion shift might be highly useful in the context in which many countries from all around the world are planning to start the COVID-19 vaccination process, as it would allow governmental decision makers to promote actions aimed at limiting the distribution of fake news and increasing the general public’s confidence towards vaccination. The analysis can be performed on a daily basis, in order to have a real-time overview of the stance evolution.

Possible future research directions include the development of better performing stance classification algorithms, as well as extending the analyzed period, especially given the fact that the vaccination process is expected to take a relatively long period of time.

## References

[1] Worldometer. (Dec. 9, 2020). Coronavirus Update (Live): 63,777,845 Cases and 1,477,777 Deaths From COVID-19 Virus Pandemic. Accessed: Dec. 9, 2020. [Online]. Available: https://www.worldometers.info/coronavirus/

[2] K. Chakraborty, S. Bhatia, S. Bhattacharyya, J. Platos, R. Bag, and A. E. Hassanien, “Sentiment analysis of COVID-19 tweets by deep learning classifiers—A study to show how popularity is affecting accuracy in social media,” Appl. Soft Comput., vol. 97, Dec. 2020, Art. no. 106754, doi: 10.1016/j.asoc.2020.106754. PMC7521435 33013254

[3] A. H. Alamoodi, B. B. Zaidan, A. A. Zaidan, O. S. Albahri, K. I. Mohammed, R. Q. Malik, E. M. Almahdi, M. A. Chyad, Z. Tareq, A. S. Albahri, H. Hameed, and M. Alaa, “Sentiment analysis and its applications in fighting COVID-19 and infectious diseases: A systematic review,” Expert Syst. Appl., vol. 167, Apr. 2021, Art. no. 114155, doi: 10.1016/j.eswa.2020.114155. PMC7591875 33139966

[4] G. Appel, L. Grewal, R. Hadi, and A. T. Stephen, “The future of social media in marketing,” J. Acad. Marketing Sci., vol. 48, no. 1, pp. 79–95, Jan. 2020, doi: 10.1007/s11747-019-00695-1. PMC7222052 32431463

[5] E. D’Andrea, P. Ducange, A. Bechini, A. Renda, and F. Marcelloni, “Monitoring the public opinion about the vaccination topic from tweets analysis,” Expert Syst. Appl., vol. 116, pp. 209–226, Feb. 2019, doi: 10.1016/j.eswa.2018.09.009.

[6] A. Giachanou and F. Crestani, “Like it or not: A survey of Twitter sentiment analysis methods,” ACM Comput. Surv., vol. 49, no. 2, Nov. 2016, Art. no. 28, doi: 10.1145/2938640.

[7] J. Golbeck, J. M. Grimes, and A. Rogers, “Twitter use by the U.S. Congress,” J. Amer. Soc. Inf. Sci. Technol., vol. 61, no. 8, pp. 1612–1621, 5 2010, doi: 10.1002/asi.21344.

[8] N. Öztürk and S. Ayvaz, “Sentiment analysis on Twitter: A text mining approach to the syrian refugee crisis,” Telematics Informat., vol. 35, no. 1, pp. 136–147, Apr. 2018, doi: 10.1016/j.tele.2017.10.006.

[9] G. A. Ruz, P. A. Henríquez, and A. Mascareño, “Sentiment analysis of Twitter data during critical events through Bayesian networks classifiers,” Future Gener. Comput. Syst., vol. 106, pp. 92–104, 5 2020, doi: 10.1016/j.future.2020.01.005.

[10] P. Tiwari, P. Yadav, S. Kumar, B. K. Mishra, G. N. Nguyen, S. P. Gochhayat, J. Singhk, and M. Prasad, “Sentiment analysis for airlines services based on Twitter dataset,” in Social Network Analytics, N. Dey, S. Borah, R. Babo, and A. S. Ashour, Eds. New York, NY, USA: Academic, 2019, pp. 149–162.

[11] N. A. Vidya, M. I. Fanany, and I. Budi, “Twitter sentiment to analyze net brand reputation of mobile phone providers,” Procedia Comput. Sci., vol. 72, pp. 519–526, Jan. 2015, doi: 10.1016/j.procs.2015.12.159.

[12] Y. Yu and X. Wang, “World cup 2014 in the Twitter world: A big data analysis of sentiments in U.S. Sports fans’ tweets,” Comput. Hum. Behav., vol. 48, pp. 392–400, Jul. 2015, doi: 10.1016/j.chb.2015.01.075.

[13] R. P. Schumaker, A. T. Jarmoszko, and C. S. Labedz, “Predicting wins and spread in the premier league using a sentiment analysis of Twitter,” Decis. Support Syst., vol. 88, pp. 76–84, Aug. 2016, doi: 10.1016/j.dss.2016.05.010.

[14] O. Kraaijeveld and J. De Smedt, “The predictive power of public Twitter sentiment for forecasting cryptocurrency prices,” J. Int. Financial Markets, Inst. Money, vol. 65, Mar. 2020, Art. no. 101188, doi: 10.1016/j.intfin.2020.101188.

[15] P.-F. Pai and C.-H. Liu, “Predicting vehicle sales by sentiment analysis of Twitter data and stock market values,” IEEE Access, vol. 6, pp. 57655–57662, 2018, doi: 10.1109/ACCESS.2018.2873730.

[16] A. Khatua, A. Khatua, and E. Cambria, “Predicting political sentiments of voters from Twitter in multi-party contexts,” Appl. Soft Comput., vol. 97, Dec. 2020, Art. no. 106743, doi: 10.1016/j.asoc.2020.106743.

[17] Y. Pershad, P. Hangge, H. Albadawi, and R. Oklu, “Social medicine: Twitter in healthcare,” J. Clin. Med., vol. 7, no. 6, p. 121, 5 2018, doi: 10.3390/jcm7060121. PMC6025547 29843360

[18] R. Kullar, D. A. Goff, T. P. Gauthier, and T. C. Smith, “To tweet or not to tweet—A review of the viral power of Twitter for infectious diseases,” Current Infectious Disease Rep., vol. 22, no. 6, Apr. 2020, Art. no. 14, doi: 10.1007/s11908-020-00723-0. PMC7180651 38624315

[19] C. M. Trovato, M. Montuori, S. Oliva, S. Cucchiara, A. Cignarelli, and A. Sansone, “Assessment of public perceptions and concerns of celiac disease: A Twitter-based sentiment analysis study,” Digestive Liver Disease, vol. 52, no. 4, pp. 464–466, Apr. 2020, doi: 10.1016/j.dld.2020.02.004. 32127326

[20] W. C. Crannell, E. Clark, C. Jones, T. A. James, and J. Moore, “A pattern-matched Twitter analysis of US cancer-patient sentiments,” J. Surgical Res., vol. 206, no. 2, pp. 536–542, Dec. 2016, doi: 10.1016/j.jss.2016.06.050. 27523257

[21] E. A. Pedersen, L. H. Loft, S. U. Jacobsen, B. Søborg, and J. Bigaard, “Strategic health communication on social media: Insights from a Danish social media campaign to address HPV vaccination hesitancy,” Vaccine, vol. 38, no. 31, pp. 4909–4915, Jun. 2020, doi: 10.1016/j.vaccine.2020.05.061. 32482460

[22] L. H. Loft, E. A. Pedersen, S. U. Jacobsen, B. Søborg, and J. Bigaard, “Using facebook to increase coverage of HPV vaccination among Danish girls: An assessment of a Danish social media campaign,” Vaccine, vol. 38, no. 31, pp. 4901–4908, Jun. 2020, doi: 10.1016/j.vaccine.2020.04.032. 32362529

[23] K. Dedominicis, A. M. Buttenheim, A. C. Howa, P. L. Delamater, D. Salmon, S. B. Omer, and N. P. Klein, “Shouting at each other into the void: A linguistic network analysis of vaccine hesitance and support in online discourse regarding California law SB277,” Social Sci. Med., vol. 266, Dec. 2020, Art. no. 113216, doi: 10.1016/j.socscimed.2020.113216. 33126093

[24] S. Martin, E. Kilich, S. Dada, P. E. Kummervold, C. Denny, P. Paterson, and H. J. Larson, “Vaccines for pregnant women···?! Absurd’—Mapping maternal vaccination discourse and stance on social media over six months,” Vaccine, vol. 38, no. 42, pp. 6627–6637, Sep. 2020, doi: 10.1016/j.vaccine.2020.07.072. 32788136

[25] T. T. Le, Z. Andreadakis, A. Kumar, R. G. Román, S. Tollefsen, M. Saville, and S. Mayhew, “The COVID-19 vaccine development landscape,” Nature Rev. Drug Discovery, vol. 19, no. 5, pp. 305–306, Apr. 2020, doi: 10.1038/d41573-020-00073-5. 32273591

[26] J. F. Modlin, W. A. Orenstein, and A. D. Brandling-Bennett, “Current status of mumps in the united states,” J. Infectious Diseases, vol. 132, no. 1, pp. 106–109, Jul. 1975. 1151120 10.1093/infdis/132.1.106

[27] B. Liu, Sentiment Analysis: Mining Opinions, Sentiments, and Emotions, 1st ed. Cambridge, U.K.: Cambridge Univ. Press, 2015.

[28] B. Liu, Sentiment Analysis and Opinion Mining, vol. 5. San Rafael, CA, USA: Morgan & Claypool, 2012.

[29] L.-A. Cotfas, C. Delcea, A. Segault, and I. Roxin, “Semantic Web-based social media analysis,” in Transactions on Computational Collective Intelligence XXII, vol. 9655, N. T. Nguyen and R. Kowalczyk, Eds. Berlin, Germany: Springer, 2016, pp. 147–166.

[30] I. Chaturvedi, E. Ragusa, P. Gastaldo, R. Zunino, and E. Cambria, “Bayesian network based extreme learning machine for subjectivity detection,” J. Franklin Inst., vol. 355, no. 4, pp. 1780–1797, Mar. 2018, doi: 10.1016/j.jfranklin.2017.06.007.

[31] R. Wang, D. Zhou, M. Jiang, J. Si, and Y. Yang, “A survey on opinion mining: From stance to product aspect,” IEEE Access, vol. 7, pp. 41101–41124, 2019, doi: 10.1109/ACCESS.2019.2906754.

[32] K. S. Hasan and V. Ng, “Extra-linguistic constraints on stance recognition in ideological debates,” in Proc. 51st Annu. Meeting Assoc. Comput. Linguistics, Sofia, Bulgaria, vol. 2, Aug. 2013, pp. 816–821. Accessed: Dec. 9, 2020. [Online]. Available: https://www.aclweb.org/anthology/P13-2142

[33] L.-A. Cotfas, C. Delcea, and I. Roxin, “Grey sentiment analysis using multiple lexicons,” in Proc. 15th Int. Conf. Informat. Economy (IE), Cluj-Napoca, Romania, 2016, pp. 428–433.

[34] L.-A. Cotfas, C. Delcea, and I. Nica, “Analysing customers’ opinions towards product characteristics using social media,” in Eurasian Business Perspectives. Cham, Switzerland: Springer, Jun. 2020, pp. 129–138, doi: 10.1007/978-3-030-48505-4_9.

[35] S. Aloufi and A. E. Saddik, “Sentiment identification in football-specific tweets,” IEEE Access, vol. 6, pp. 78609–78621, Dec. 2018, doi: 10.1109/ACCESS.2018.2885117.

[36] E. Cambria, Y. Li, F. Z. Xing, S. Poria, and K. Kwok, “SenticNet 6: Ensemble application of symbolic and subsymbolic AI for sentiment analysis,” in Proc. 29th ACM Int. Conf. Inf. Knowl. Manage., New York, NY, USA, Oct. 2020, pp. 105–114, doi: 10.1145/3340531.3412003.

[37] M. Hu and B. Liu, “Mining and summarizing customer reviews,” in Proc. ACM SIGKDD Int. Conf. Knowl. Discovery Data Mining (KDD), New York, NY, USA, 2004, pp. 168–177, doi: 10.1145/1014052.1014073.

[38] S. Kiritchenko, X. Zhu, and S. M. Mohammad, “Sentiment analysis of short informal texts,” J. Artif. Intell. Res., vol. 50, pp. 723–762, Aug. 2014, doi: 10.1613/jair.4272.

[39] S. Mohammad, S. Kiritchenko, and X. Zhu, “NRC-Canada: Building the state-of-the-art in sentiment analysis of tweets,” in Proc. 2nd Joint Conf. Lexical Comput. Semantics (SEM), 7th Int. Workshop Semantic Eval. (SemEval), Atlanta, GA, USA, vol. 2, Jun. 2013, pp. 321–327. Accessed: Dec. 9, 2020. [Online]. Available: https://www.aclweb.org/anthology/S13-2053

[40] C. J. Hutto and E. Gilbert, “VADER: A parsimonious rule-based model for sentiment analysis of social media text,” presented at the 8th Int. AAAI Conf. Weblogs Social Media, Ann Arbor, MI, USA, May 2014. Accessed: Nov. 8, 2020. [Online]. Available: http://www.aaai.org/ocs/index.php/ICWSM/ICWSM14/paper/view/8109

[41] S. Baccianella, A. Esuli, and F. Sebastiani, “SentiWordNet 3.0: An enhanced lexical resource for sentiment analysis and opinion mining,” in Proc. LREC, vol. 10, 2010, pp. 2200–2204. Accessed: Nov. 10, 2020. [Online]. Available: http://lrec-conf.org/proceedings/lrec2010/pdf/769_Paper.pdf

[42] E. Cambria, C. Havasi, and A. Hussain, “SenticNet 2: A semantic and affective resource for opinion mining and sentiment analysis,” presented at the 25h Int. Florida Artif. Intell. Res. Soc. Conf., Marco Island, FL, USA, May 2012.

[43] A. Dey, M. Jenamani, and J. J. Thakkar, “Senti-N-Gram: An n-gram lexicon for sentiment analysis,” Expert Syst. Appl., vol. 103, pp. 92–105, Aug. 2018, doi: 10.1016/j.eswa.2018.03.004.

[44] N. Mukhtar, M. A. Khan, and N. Chiragh, “Lexicon-based approach outperforms supervised machine learning approach for urdu sentiment analysis in multiple domains,” Telematics Informat., vol. 35, no. 8, pp. 2173–2183, Dec. 2018, doi: 10.1016/j.tele.2018.08.003.

[45] J. Brownlee, Deep Learning for Natural Language Processing: Develop Deep Learning Models for Your Natural Language Problems. Vermont, VIC, USA: Machine Learning Mastery, 2017.

[46] I. Goodfellow, Y. Bengio, and A. Courville, Deep Learning. Cambridge, MA, USA: MIT Press, 2016.

[47] T. Mikolov, I. Sutskever, K. Chen, G. Corrado, and J. Dean, “Distributed representations of words and phrases and their compositionality,” in Proc. 26th Int. Conf. Neural Inf. Process. Syst., Red Hook, NY, USA, vol. 2, Dec. 2013, pp. 3111–3119. Accessed: Dec. 10, 2020.

[48] J. Pennington, R. Socher, and C. Manning, “Glove: Global vectors for word representation,” in Proc. Conf. Empirical Methods Natural Lang. Process. (EMNLP), Doha, Qatar, Oct. 2014, pp. 1532–1543, doi: 10.3115/v1/D14-1162.

[49] A. Joulin, E. Grave, P. Bojanowski, and T. Mikolov, “Bag of tricks for efficient text classification,” in Proc. 15th Conf. Eur. Chapter Assoc. Comput. Linguistics, Valencia, Spain, vol. 2, Apr. 2017, pp. 427–431. Accessed: Feb. 6, 2021. [Online]. Available: https://www.aclweb.org/anthology/E17-2068

[50] K. Dey, R. Shrivastava, and S. Kaushik, “Twitter stance detection—A subjectivity and sentiment polarity inspired two-phase approach,” in Proc. IEEE Int. Conf. Data Mining Workshops (ICDMW), Nov. 2017, pp. 365–372, doi: 10.1109/ICDMW.2017.53.

[51] S. Mohammad, S. Kiritchenko, P. Sobhani, X. Zhu, and C. Cherry, “SemEval-2016 task 6: Detecting stance in tweets,” in Proc. 10th Int. Workshop Semantic Eval. (SemEval), San Diego, CA, USA, 2016, pp. 31–41, doi: 10.18653/v1/S16-1003.

[52] L. Zhang, S. Wang, and B. Liu, “Deep learning for sentiment analysis: A survey,” Wiley Interdiscipl. Rev., Data Mining Knowl. Discovery, vol. 8, no. 4, Jul. 2018, Art. no. e1253, doi: 10.1002/widm.1253.

[53] N. Kalchbrenner, E. Grefenstette, and P. Blunsom, “A convolutional neural network for modelling sentences,” in Proc. 52nd Annu. Meeting Assoc. Comput. Linguistics, Baltimore, MD, USA, 2014, pp. 655–665, doi: 10.3115/v1/P14-1062.

[54] T. Mikolov, M. Karafiát, L. Burget, and S. Khudanpur, “Recurrent neural network based language model,” in Proc. INTERSPEECH, Japan, Sep. 2010, pp. 1045–1048. [Online]. Available: https://www.isca-speech.org/archive/archive_papers/interspeech_2010/i10_1045.pdf

[55] A. Benton and M. Dredze, “Using author embeddings to improve tweet stance classification,” in Proc. 4th Workshop Noisy User-Gener. Text EMNLP Workshop (W-NUT), Brussels, Belgium, Nov. 2018, pp. 184–194, doi: 10.18653/v1/W18-6124.

[56] Q. Sun, Z. Wang, Q. Zhu, and G. Zhou, “Stance detection with hierarchical attention network,” in Proc. 27th Int. Conf. Comput. Linguistics, Santa Fe, NM, USA, Aug. 2018, pp. 2399–2409. Accessed: Dec. 9, 2020. [Online]. Available: https://www.aclweb.org/anthology/C18-1203

[57] J. Du, R. Xu, Y. He, and L. Gui, “Stance classification with target-specific neural attention networks,” in Proc. 26th Int. Joint Conf. Artif. Intell. (IJCAI), Int. Joint Conf. Artif. Intell. (AUS), 2017, pp. 3988–3994.

[58] G. Zarrella and A. Marsh, “MITRE at SemEval-2016 task 6: Transfer learning for stance detection,” in Proc. 10th Int. Workshop Semantic Eval. (SemEval), San Diego, CA, USA, Jun. 2016, pp. 458–463, doi: 10.18653/v1/S16-1074.

[59] A. Vaswani, N. Shazeer, N. Parmar, J. Uszkoreit, L. Jones, A. N. Gomez, L. Kaiser, and I. Polosukhin, “Attention is all you need,” in Proc. 31st Int. Conf. Neural Inf., Long Beach, CA, USA, Dec. 2017, pp. 6000–6010. Accessed: Jan. 24, 2021. [Online]. Available: https://dl.acm.org/doi/pdf/10.5555/3295222.3295349

[60] P. J. Liu, M. Saleh, E. Pot, B. Goodrich, R. Sepassi, L. Kaiser, and N. Shazeer, “Generating wikipedia by summarizing long sequences,” presented at the 6th Int. Conf. Learn. Represent., Apr. 2018. [Online]. Available: https://openreview.net/forum?id=Hyg0vbWC-

[61] N. Kitaev and D. Klein, “Constituency parsing with a self-attentive encoder,” in Proc. 56th Annu. Meeting Assoc. Comput. Linguistics, Melbourne, VIC, Australia, Jul. 2018, pp. 2676–2686, doi: 10.18653/v1/P18-1249.

[62] A. Radford, K. Narasimhan, T. Salimans, and I. Sutskever. (2018). Improving Language Understanding by Generative Pre-Training. [Online]. Available: https://cdn.openai.com/research-covers/language-unsupervised/language_understanding_paper.pdf

[63] J. Devlin, M.-W. Chang, K. Lee, and K. Toutanova, “BERT: Pre-training of deep bidirectional transformers for language understanding,” in Proc. Conf. North Amer. Chapter Assoc. Comput. Linguistics, Hum. Lang. Technol., Minneapolis, MN, USA, Jun. 2019, pp. 4171–4186, doi: 10.18653/v1/N19-1423.

[64] R. M. Merchant and N. Lurie, “Social media and emergency preparedness in response to novel coronavirus,” J. Amer. Med. Assoc., vol. 323, no. 20, pp. 2011–2012, 5 2020, doi: 10.1001/jama.2020.4469. 32202611

[65] S. Kaur, P. Kaul, and P. M. Zadeh, “Monitoring the dynamics of emotions during COVID-19 using Twitter data,” Procedia Comput. Sci., vol. 177, pp. 423–430, Jan. 2020, doi: 10.1016/j.procs.2020.10.056.

[66] P. Singh, S. Singh, M. Sohal, Y. K. Dwivedi, K. S. Kahlon, and R. S. Sawhney, “Psychological fear and anxiety caused by COVID-19: Insights from Twitter analytics,” Asian J. Psychiatry, vol. 54, Dec. 2020, Art. no. 102280, doi: 10.1016/j.ajp.2020.102280. PMC7836781 32688277

[67] S. Boon-Itt and Y. Skunkan, “Public perception of the COVID-19 pandemic on Twitter: Sentiment analysis and topic modeling study,” JMIR Public Health Surveill., vol. 6, no. 4, Nov. 2020, Art. no. e21978, doi: 10.2196/21978. PMC7661106 33108310

[68] J. Xue, J. Chen, C. Chen, C. Zheng, S. Li, and T. Zhu, “Public discourse and sentiment during the COVID 19 pandemic: Using latent Dirichlet allocation for topic modeling on Twitter,” PLoS ONE, vol. 15, no. 9, Sep. 2020, Art. no. e0239441, doi: 10.1371/journal.pone.0239441. PMC7518625 32976519

[69] M. Bhat, M. Qadri, N.-U.-A. Beg, M. Kundroo, N. Ahanger, and B. Agarwal, “Sentiment analysis of social media response on the COVID19 outbreak,” Brain, Behav., Immunity, vol. 87, pp. 136–137, Jul. 2020, doi: 10.1016/j.bbi.2020.05.006. PMC7207131 32418721

[70] A. Kruspe, M. Häberle, I. Kuhn, and X. X. Zhu, “Cross-language sentiment analysis of European Twitter messages during the COVID-19 pandemic,” presented at the ACL-NLP-COVID, Jul. 2020. Accessed: Feb. 6, 2021. [Online]. Available: https://www.aclweb.org/anthology/2020.nlpcovid19-acl.14

[71] B. P. Pokharel, “Twitter sentiment analysis during COVID-19 outbreak in nepal,” Social Sci. Res. Netw., Rochester, NY, USA, Tech. Rep. 3624719, Jun. 2020, doi: 10.2139/ssrn.3624719.

[72] G. Barkur, Vibha, and G. B. Kamath, “Sentiment analysis of nationwide lockdown due to COVID 19 outbreak: Evidence from india,” Asian J. Psychiatry, vol. 51, Jun. 2020, Art. no. 102089, doi: 10.1016/j.ajp.2020.102089. PMC7152888 32305035

[73] R. Khan, P. Shrivastava, A. Kapoor, A. Tiwari, and A. Mittal, “Social media analysis with AI: Sentiment analysis techniques for the analysis of Twitter COVID-19 Data,” J. Crit. Rev., vol. 7, no. 9, pp. 2761–2774, 2020.

[74] A. S. Imran, S. M. Daudpota, Z. Kastrati, and R. Batra, “Cross-cultural polarity and emotion detection using sentiment analysis and deep learning on COVID-19 related tweets,” IEEE Access, vol. 8, pp. 181074–181090, Sep. 2020, doi: 10.1109/ACCESS.2020.3027350. PMC8545282 34812358

[75] J. Samuel, G. G. M. N. Ali, M. M. Rahman, E. Esawi, and Y. Samuel, “COVID-19 public sentiment insights and machine learning for tweets classification,” Information, vol. 11, no. 6, p. 314, Jun. 2020, doi: 10.3390/info11060314.

[76] J. Zhou, S. Yang, C. Xiao, and F. Chen, “Examination of community sentiment dynamics due to COVID-19 pandemic: A case study from Australia,” Jun. 2020, arXiv:2006.12185. Accessed: Dec. 3, 2020. [Online]. Available: http://arxiv.org/abs/2006.12185 10.1007/s42979-021-00596-7 PMC8034046 33851137

[77] X. Wang, C. Zou, Z. Xie, and D. Li, “Public Opinions towards COVID-19 in California and New York on Twitter,” medRxiv, Jul. 2020, doi: 10.1101/2020.07.12.20151936.

[78] C. K. Pastor, “Sentiment analysis of filipinos and effects of extreme community quarantine due to coronavirus (COVID-19) pandemic,” J. Crit. Rev., vol. 7, no. 7, pp. 91–95, Apr. 2020, doi: 10.31838/jcr.07.07.15.

[79] A. Sanders, R. White, L. Severson, R. Ma, R. McQueen, H. C. A. Paulo, Y. Zhang, J. S. Erickson, and K. P. Bennett, “Unmasking the conversation on masks: Natural language processing for topical sentiment analysis of COVID-19 Twitter discourse,” medRxiv, Sep. 2020, doi: 10.1101/2020.08.28.20183863. PMC8378598 34457171

[80] Y. Zhang, H. Lyu, Y. Liu, X. Zhang, Y. Wang, and J. Luo, “Monitoring depression trend on Twitter during the COVID-19 pandemic,” Jul. 2020, arXiv:2007.00228. Accessed: Dec. 3, 2020. [Online]. Available: http://arxiv.org/abs/2007.00228

[81] Y. Lu and Q. Zheng, “Twitter public sentiment dynamics on cruise tourism during the COVID-19 pandemic,” Current Issues Tourism, pp. 1–7, Nov. 2020, doi: 10.1080/13683500.2020.1843607.

[82] J. Xue, J. Chen, R. Hu, C. Chen, C. Zheng, Y. Su, and T. Zhu, “Twitter discussions and emotions about the COVID-19 pandemic: Machine learning approach,” J. Med. Internet Res., vol. 22, no. 11, Nov. 2020, Art. no. e20550, doi: 10.2196/20550. PMC7690968 33119535

[83] S. Noor, Y. Guo, S. H. H. Shah, P. Fournier-Viger, and M. S. Nawaz, “Analysis of public reactions to the novel Coronavirus (COVID-19) outbreak on Twitter,” Kybernetes, 2020, doi: 10.1108/K-05-2020-0258.

[84] M. K. Elhadad, K. F. Li, and F. Gebali, “COVID-19-FAKES: A Twitter (Arabic/English) dataset for detecting misleading information on COVID-19,” in Advances in Intelligent Networking and Collaborative Systems, vol. 1263. Cham, Switzerland: Springer, Aug. 2020, pp. 256–268, doi: 10.1007/978-3-030-57796-4_25.

[85] R. K. Gupta, A. Vishwanath, and Y. Yang, “COVID-19 Twitter dataset with latent topics, sentiments and emotions attributes,” Sep. 2020, arXiv:2007.06954. Accessed: Nov. 12, 2020. [Online]. Available: http://arxiv.org/abs/2007.06954

[86] J. M. Banda, R. Tekumalla, G. Wang, J. Yu, T. Liu, Y. Ding, K. Artemova, E. Tutubalina, and G. Chowell, “A large-scale COVID-19 Twitter chatter dataset for open scientific research—An international collaboration,” Nov. 2020, arXiv:2004.03688. Accessed: Dec. 1, 2020. [Online]. Available: http://arxiv.org/abs/2004.03688 10.3390/epidemiologia2030024 PMC9620940 36417228

[87] R. Lamsal, “Design and analysis of a large-scale COVID-19 Tweets dataset,” Appl. Intell., 2020, doi: 10.1007/s10489-020-02029-z. PMC7646503 34764561

[88] E. Chen, K. Lerman, and E. Ferrara, “Tracking social media discourse about the COVID-19 pandemic: Development of a public coronavirus Twitter data set,” JMIR Public Health Surveill., vol. 6, no. 2, 5 2020, Art. no. e19273, doi: 10.2196/19273. PMC7265654 32427106

[89] S. Mohammad, S. Kiritchenko, P. Sobhani, X. Zhu, and C. Cherry, “A dataset for detecting stance in tweets,” in Proc. 10th Int. Conf. Lang. Resour. Eval. (LREC), Portorož, Slovenia, May 2016, pp. 3945–3952. Accessed: Dec. 11, 2020. [Online]. Available: https://www.aclweb.org/anthology/L16-1623

[90] Y. Bao, C. Quan, L. Wang, and F. Ren, “The role of pre-processing in Twitter sentiment analysis,” in Intelligent Computing Methodologies, vol. 8589. Cham, Switzerland: Springer, 2014, pp. 615–624, doi: 10.1007/978-3-319-09339-0_62.

[91] Z. Jianqiang and G. Xiaolin, “Comparison research on text pre-processing methods on Twitter sentiment analysis,” IEEE Access, vol. 5, pp. 2870–2879, Feb. 2017, doi: 10.1109/ACCESS.2017.2672677.

[92] C. Baziotis, N. Pelekis, and C. Doulkeridis, “DataStories at SemEval-2017 task 4: Deep LSTM with attention for message-level and topic-based sentiment analysis,” in Proc. 11th Int. Workshop Semantic Eval. (SemEval), Vancouver, BC, Canada, Aug. 2017, pp. 747–754, doi: 10.18653/v1/S17-2126.

[93] S. Bird, E. Klein, and E. Loper, Natural Language Processing With Python: Analyzing Text With the Natural Language Toolkit, 1st ed. Beijing, China: O’Reilly Media, 2009.

[94] F. C. Peng and D. Schuurmans, “Combining naive Bayes and n-gram language models for text classification,” in Advances in Information Retrieval, vol. 2633. Berlin, Germany: Springer, Apr. 2003, pp. 335–350.

[95] M.-L. Zhang, J. M. Peña, and V. Robles, “Feature selection for multi-label naive Bayes classification,” Inf. Sci., vol. 179, no. 19, pp. 3218–3229, Sep. 2009, doi: 10.1016/j.ins.2009.06.010.

[96] A. McCallum, K. Nigam, and others, “A comparison of event models for naive Bayes text classification,” in Proc. AAAI Workshop Learn. Text Categorization, 1998, vol. 752, no. 1, pp. 41–48.

[97] L. Breiman, “Random forests,” Mach. Learn., vol. 45, no. 1, pp. 5–32, 2001.

[98] S. Misra and H. Li, “Noninvasive fracture characterization based on the classification of sonic wave travel times,” in Machine Learning for Subsurface Characterization, S. Misra, H. Li, and J. He, Eds. Pittsburgh, PA, USA: Gulf Professional Publishing, 2020, pp. 243–287.

[99] J. C. Platt, “Fast training of support vector machines using sequential minimal optimization,” in Advances in Kernel Methods: Support Vector Learning. Cambridge, MA, USA: MIT Press, 1999, pp. 185–208.

[100] V. Mohammadi and S. Minaei, “Artificial intelligence in the production process,” in Engineering Tools in the Beverage Industry, A. M. Grumezescu and A. M. Holban, Eds. Sawston, U.K.: Woodhead Publishing, 2019, pp. 27–63.

[101] D. Ballabio and R. Todeschini, “Multivariate classification for qualitative analysis,” in Infrared Spectroscopy for Food Quality Analysis and Control, D.-W. Sun, Ed. San Diego, CA, USA: Academic, 2009, pp. 83–104.

[102] S. Hochreiter and J. Schmidhuber, “Long short-term memory,” Neural Comput., vol. 9, no. 8, pp. 1735–1780, Nov. 1997, doi: 10.1162/neco.1997.9.8.1735. 9377276

[103] S. Rosenthal, N. Farra, and P. Nakov, “SemEval-2017 task 4: Sentiment analysis in Twitter,” in Proc. 11th Int. Workshop Semantic Eval. (SemEval), Vancouver, BC, Canada, Aug. 2017, pp. 502–518, doi: 10.18653/v1/S17-2088.

[104] M. Cliche, “BB_twtr at SemEval-2017 task 4: Twitter sentiment analysis with CNNs and LSTMs,” in Proc. 11th Int. Workshop Semantic Eval. (SemEval), Vancouver, BC, Canada, Aug. 2017, pp. 573–580, doi: 10.18653/v1/S17-2094.

[105] W. Wei, X. Zhang, X. Liu, W. Chen, and T. Wang, “pkudblab at SemEval-2016 task 6: A specific convolutional neural network system for effective stance detection,” in Proc. 10th Int. Workshop Semantic Eval. (SemEval), San Diego, CA, USA, Jun. 2016, pp. 384–388, doi: 10.18653/v1/S16-1062.

[106] C. Baziotis. (2020). Deep-Learning Model Presented in ‘DataStories at SemEval-2017 Task 4: Deep LSTM With Attention for Message-Level and Topic-based Sentiment Analysis’. [Online]. Available: https://github.com/cbaziotis/datastories-semeval2017-task4

[107] C. Chelba, T. Mikolov, M. Schuster, Q. Ge, T. Brants, P. Koehn, and T. Robinson, “One billion word benchmark for measuring progress in statistical language modeling,” in Proc. INTERSPEECH, Singapore, Sep. 2014, pp. 2635–2639. Accessed: Jan. 24, 2021. [Online]. Available: http://arxiv.org/abs/1312.3005

[108] I. Turc, M.-W. Chang, K. Lee, and K. Toutanova, “Well-read students learn better: On the importance of pre-training compact models,” Sep. 2019, arXiv:1908.08962. Accessed: Jan. 24, 2021. [Online]. Available: http://arxiv.org/abs/1908.08962

[109] F. Pedregosa, G. Varoquaux, A. Gramfort, V. Michel, B. Thirion, O. Grisel, M. Blondel, P. Prettenhofer, R. Weiss, V. Dubourg, J. Vanderplas, A. Passos, and D. Cournapeau, “Scikit-learn: Machine Learning in Python,” J. Mach. Learn. Res., vol. 12, pp. 2825–2830, Nov. 2011.

[110] S. M. Mohammad, P. Sobhani, and S. Kiritchenko, “Stance and sentiment in tweets,” ACM Trans. Internet Technol., vol. 17, no. 3, pp. 1–23, Jul. 2017.

[111] R. Parker, D. Graff, J. Kong, K. Chen, and K. Maeda, English Gigaword, 5th ed. Philadelphia, PA, USA: Linguistic Data Consortium, doi: 10.35111/WK4F-QT80.

[112] D. P. Kingma and J. Ba, “Adam: A method for stochastic optimization,” presented at the 3rd Int. Conf. Learn. Represent., San Diego, CA, USA, May 2015. [Online]. Available: https://arxiv.org/pdf/1412.6980.pdf

